# Volatile compounds of *Bacillus pseudomycoides* induce growth and drought tolerance in wheat (*Triticum aestivum* L.)

**DOI:** 10.1038/s41598-022-22354-2

**Published:** 2022-11-09

**Authors:** Gobindo Kumar Paul, Shafi Mahmud, Amit Kumar Dutta, Swagotom Sarkar, Aysha Akter Laboni, Md. Shamim Hossain, Abir Nagata, Pranab Karmaker, Mamudul Hasan Razu, Taheruzzaman Kazi, Md. Salah Uddin, Shahriar Zaman, Md Sayeedul Islam, Mala Khan, Md. Abu Saleh

**Affiliations:** 1 Microbiology Laboratory, Department of Genetic Engineering and Biotechnology, University of Rajshahi, Rajshahi, 6205 Bangladesh; 2 Division of Genome Sciences and Cancer, The John Curtin School of Medical Research, and The Shine-Dalgarno Centre for RNA Innovation, The Australian National University, Canberra, ACT 2601 Australia; 3 Department of Microbiology, University of Rajshahi, Rajshahi, 6205 Bangladesh; 4 Bangladesh Reference Institute for Chemical Measurements (BRiCM), Dhaka, Bangladesh; 5 Department of Biotechnology and Genetic Engineering, Islamic University, Kushtia, 7003 Bangladesh; 6 Department of Regenerative Dermatology, Graduate School of Medicine, Osaka University, Suita, 565-0871 Japan; 7 Department of Biological Sciences, Graduate School of Science, Osaka University, Machikaneyama-Cho 1-1, Toyonaka, Osaka 560-0043 Japan

**Keywords:** Biotechnology, Microbiology, Plant sciences

## Abstract

The plant growth-boosting biofilm-forming bacteria *Bacillus pseudomycoides* is able to promote growth and drought stress tolerance in wheat by suppressing the MYB gene, which synthesizes Myb protein (TaMpc1-D4) through secreted volatile compounds. In the present study, *Triticum aestivum* seeds were inoculated with five distinct bacterial strains. The growth, germination rate, root-shoot length, RWC, and chlorophyll content of seedlings were investigated. Furthermore, the levels of soluble sugars, proteins, H_2_O_2_, NO, cell death, and antioxidant enzymes (CAT, SOD, POD, and APX) were observed throughout the growth stage. All of the results showed that *B. pseudomycoides* had a substantially higher ability to form biofilm and promote these traits than the other strains. In terms of molecular gene expression, *B. pseudomycoides* inoculation strongly expressed the Dreb1 gene by silencing the expression of MYB gene through secreted volatile compounds. For identifying the specific volatile compound that silenced the MYB gene, molecular docking with Myb protein was performed. Out of 45 volatile compounds found, 2,6-ditert-butylcyclohexa-2,5-diene-1,4-dione and 3,5-ditert-butylphenol had a binding free energy of − 6.2 and − 6.5, Kcal/mol, respectively, which predicted that these compounds could suppress this protein's expression. In molecular dynamics simulations, the RMSD, SASA, Rg, RMSF, and hydrogen bonding values found assured the docked complexes' binding stability. These findings suggest that these targeted compounds may be suppressing Myb protein expression as well as the expression of Dreb1 and other drought response genes in wheat. More research (field trial) into plant growth and drought stress is needed to support the findings of this study.

## Introduction

*Triticum aestivum* is the principal source of food for most of the world’s population including many American, European, Australian, African, and some Asian countries^1,2^. It is the main component of foods such as bread, biscuits, pasta, noodles, cakes, pizza, beer, and vodka, and contains carbohydrates (71%), protein (13%), water (13%), fat (1.5%), and small amounts of phosphorus, niacin, and dietary fiber^1,3^. It is estimated that the world population will exceed 8 billion in 2030, and ensuring food security for such a large population will be a major issue for the agricultural sector due to water scarcity^4^. Future drought mitigation gains are critical if food demand for the vast population is to be met^5^. Drought intensity is growing as a result of climate change and rising temperatures around the world, causing agricultural crops to suffer^6^. In Bangladesh, for instance, most of the farmers in the 13,000 sq. km Barind Tract land in the Districts of Rajshahi, Dinajpur, and Bogura face water scarcity.

Some beneficial microorganisms influence plant metabolism to help plants survive from abiotic stress conditions such as drought and salt stress^7^. Bacterial species in genera such as *Bacillus, Enterobacter, Azospirillum, Klebsiella, Pseudomonas, Arthobacter,* and *Serratia*, among others, enrich growth by directly or indirectly supplying nutrients^8–10^, while secretion of indole acetic acid and cytokinin-like phytohormones promotes root area and surface proliferation^11^. Apart from this, cytokinins are also involved in the management of environmental stresses by transfer signaling from root to shoot^12^. Drought causes the production of reactive oxygen species (ROS), which can harm numerous plant structures^13^, such as proteins and lipids, and cause nucleic acid breakdown^14^. Plant cells include many antioxidant enzymes such as APX (ascorbate peroxidase), CAT (catalase), SOD (superoxide dismutase), POD (peroxides), and GPX (glutathione peroxidase), which reduce free radical H_2_O_2_ and superoxide O^-^_2_ production during drought conditions and improve plant survival^15,16^. Under extreme drought conditions, Plant-Growth Promoting Rhizobacteria (PGPR) improved an antioxidant CAT, implying that it can reduce oxidative damage^17^.

Researchers have recently reported that bacterial biofilm formation on root hairs under drought conditions may have a substantial positive impact on plants during high levels of drought stress^18–20^. Therefore, we chose five bacterial strains (*Bacillus pseudomycoides*, *Bacillus massilioanrexius*, *Bacillus thuringiensis*, *Serratia marcescens,* and *Acinetobacter* sp.), although only *B. pseudomycoides* has been shown to have potentially to produce biofilms in dry conditions. Volatile compounds (VOCs) are organic molecules released by bacteria that mediate increased biomass in plant roots, disease resistance, and abiotic stress tolerance^19^ and do not include any known plant growth hormones^20,21^, but they do mediate plant endogenous auxin homeostasis and iron uptake^20,22^.

Previous studies have found that over expression of the R2R3 MYB gene reduces drought tolerance and has detrimental consequences in wheat under drought stress^23^. As a result, silencing of this gene may result in increased drought tolerance. Molecular docking is a powerful method for identifying putative inhibitors based on protein–ligand interactions, with molecular simulation used to validate the binding poses and orientations. To identify the best binding compounds regarding targeted protein active regions, homology modeling of myb protein and volatile compounds were used for molecular docking. This technique is both cost- and time-effective for understanding the relationship between proteins and ligands^24,25^.

Many researchers are attempting to develop drought-tolerant wheat varieties that are both low-cost and ecofriendly in response to global climate concerns^26^. The current study aimed to find environmentally friendly bacterial strains that can form biofilms that aid in plant growth promotion. By possibly silencing the R2R3 MYB gene and increasing the expression of Dreb1 genes in wheat, specific volatile compounds that might help with drought stress tolerance could be identified.

## Results

### Morpho-physiological characteristics

**Figure 1:**
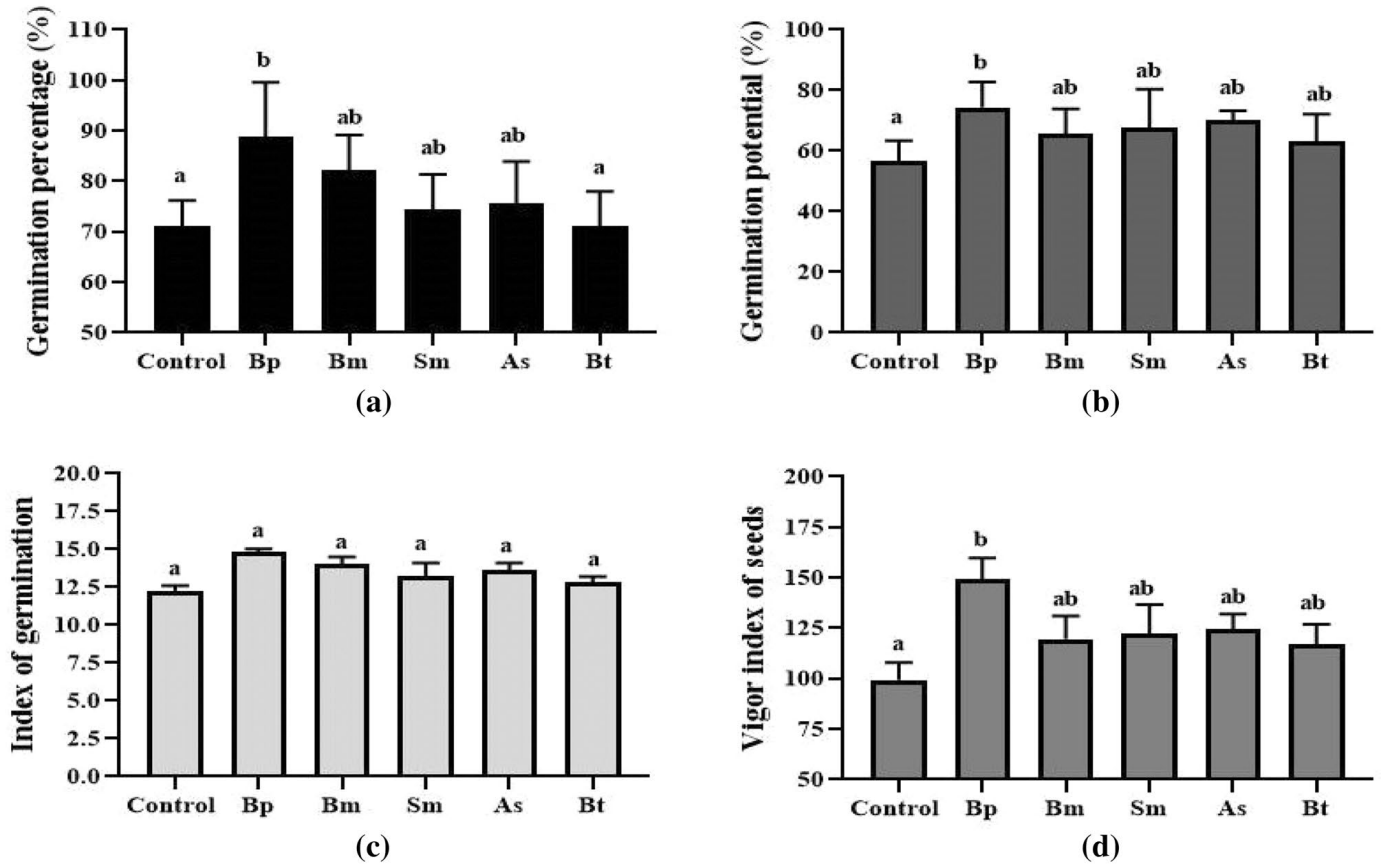
Germination parameters of wheat with five different bacterial inoculations. Here, (**a**); indicates germination rate, (**b**); indicates germination potential, (**c**); indicates germination index, and (**d**); indicates vigor index of seeds. Different letters indicate significant differences between mean ± SD of replications (n = 3) at a *P* < 0.05 significance level.

**Figure 2:**
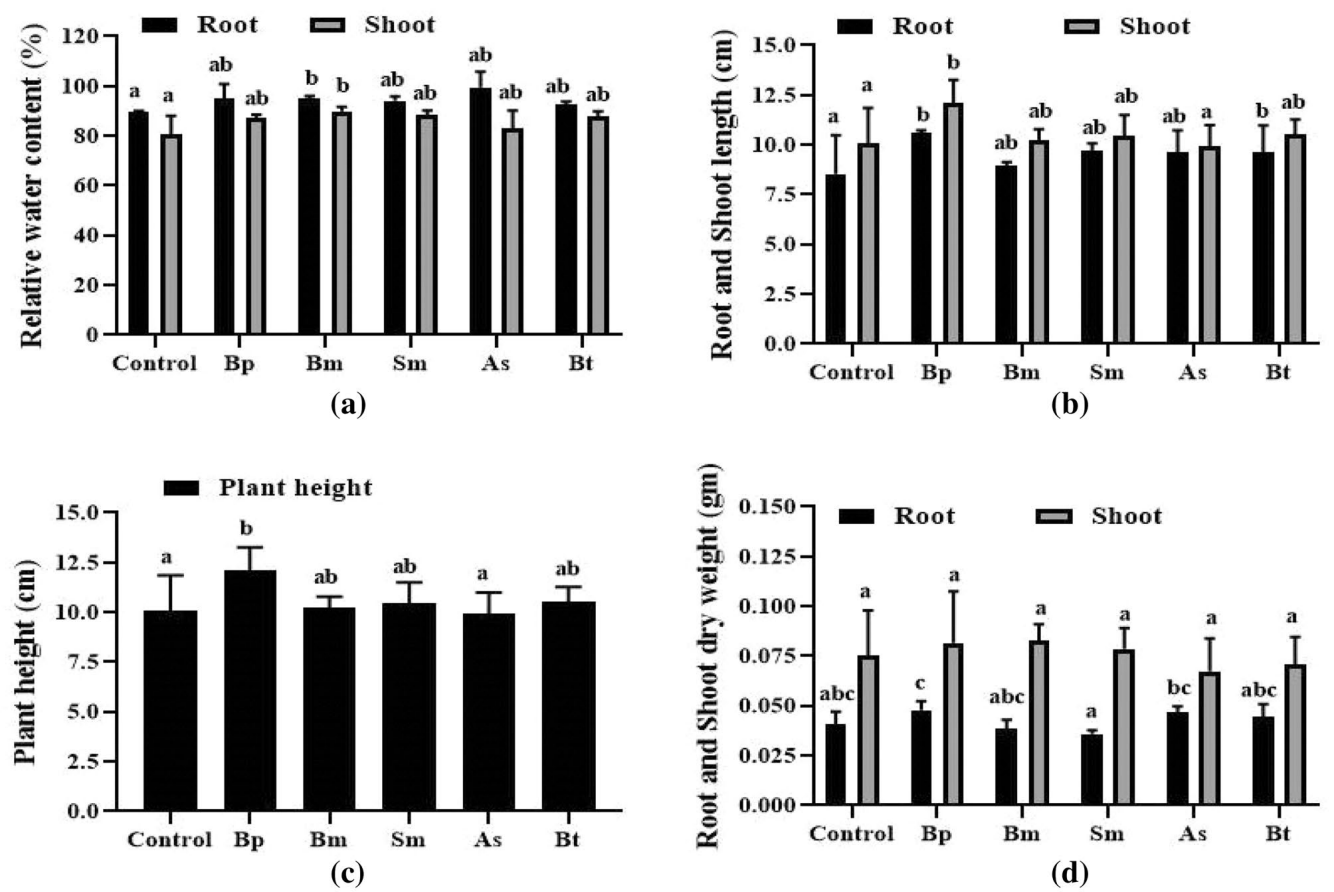
Morpho-physiological characteristics of wheat. Here, (**a**); indicates RWC content, (**b**); indicates root and shoot length, (**c**); indicates plant height and, (**d**); indicates root and shoot dry weight. Different letters indicate significant differences between mean ± SD of replications (n = 3) at a *P* < 0.05 significance level.

The Bp treatment had a germination rate of 88.8%, while Bm had an 82.22% germination rate, Sm had a 74.44% germination rate, As had a 75.55% germination rate, Bt had a 71.11% germination rate, and the Control group a 71.11% germination rate (Fig. 1a and Fig. S1). Bp inoculation demonstrated a higher germination rate when compared to other inoculations and controls. This inoculation also considerably boosted germination potential (Fig. 1b), germination index (Fig. 1c), and vigor index (Fig. 1d). When compared to wheat control, Bp had considerably increased relative water content (Fig. 2a), average root and shoot length (Fig. 2b and Fig. S2), plant height (Fig. 2c), root and shoot dry weight (Fig. 2d). In the case of Bp, the relative water content in root was 94.90% and the shoot was 87.01% (Fig. 2a) where the average root length and plant heights were 10.60 cm and 12.12 cm, respectively, whereas the control was 8.55 cm and 10.09 cm, respectively, after 10 days of inoculation (Fig. 2b). For average root-shoot dry weight, Bp was 0.048 g and 0.082 g (Fig. 2d).

### Biochemical changes

**Figure 3:**
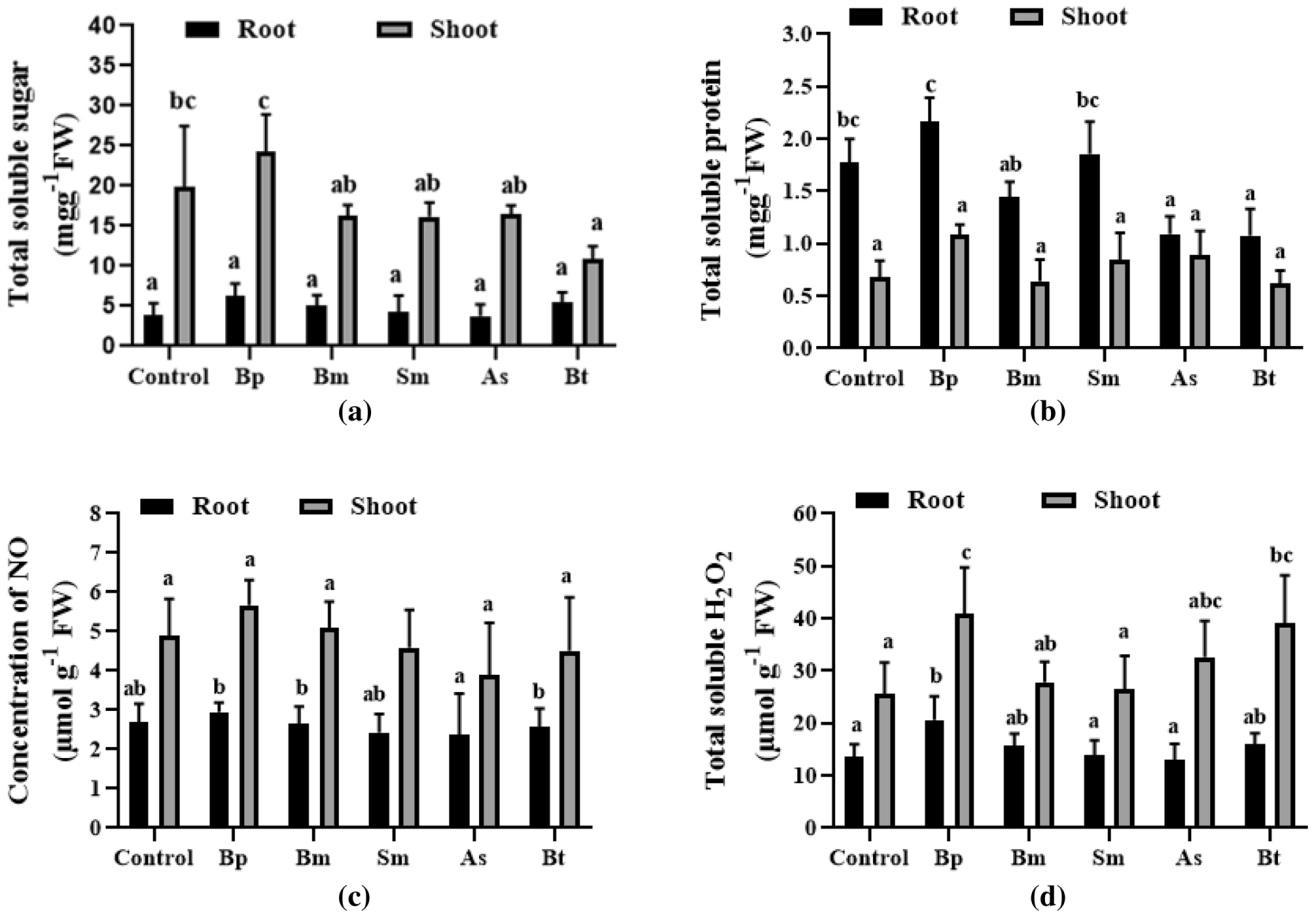
Biochemical characterization of wheat root and soot treated with five different bacterial strains. Here, (**a**), (**b**), (**c**), and (**d**) are indicating total soluble sugar, total soluble protein, NO concentration, and total soluble H_2_O_2,_ respectively in both root and shoot. Different letters indicate significant differences between mean ± SD of replications (n = 3) at a *P* < 0.05 significance level.

In the biochemical analysis, total soluble sugar and protein content in the Bp inoculation were moderately higher than control plants, although these were lower in other inoculations. Sugar concentration rose significantly in the root, averaging 6.20 mg g^−1^ and 5.08 mg g^−1^, respectively, in both Bp and Bm inoculation compared to 3.81 mg g^-1^ in the control (Fig. 3a). For the Bp inoculation, the protein levels in the root and shoot were 2.17 mg g^−1^ and 15.77 mg g^−1^, respectively, whereas the controls were 1.78 mg g^−1^ and 10.02 mg g^−1^ (Fig. 3b). Protein concentrations in the shoot, on the other hand, did not vary significantly. Furthermore, Bp inoculation resulted in higher NO and H_2_O_2_ concentrations than the other inoculations, with NO concentrations of 5.67 mg g^−1^ for the Bp inoculation and 4.89 mg g^−1^ for the control (Fig. 3c) and H_2_O_2_ concentration for the Bp inoculation of 20.63 mg g^−1^ where the control was 13.56 mg g^−1^ (Fig. 3d).

### Chlorophyll, Fe, Zn, and cell death measurement

**Figure 4:**
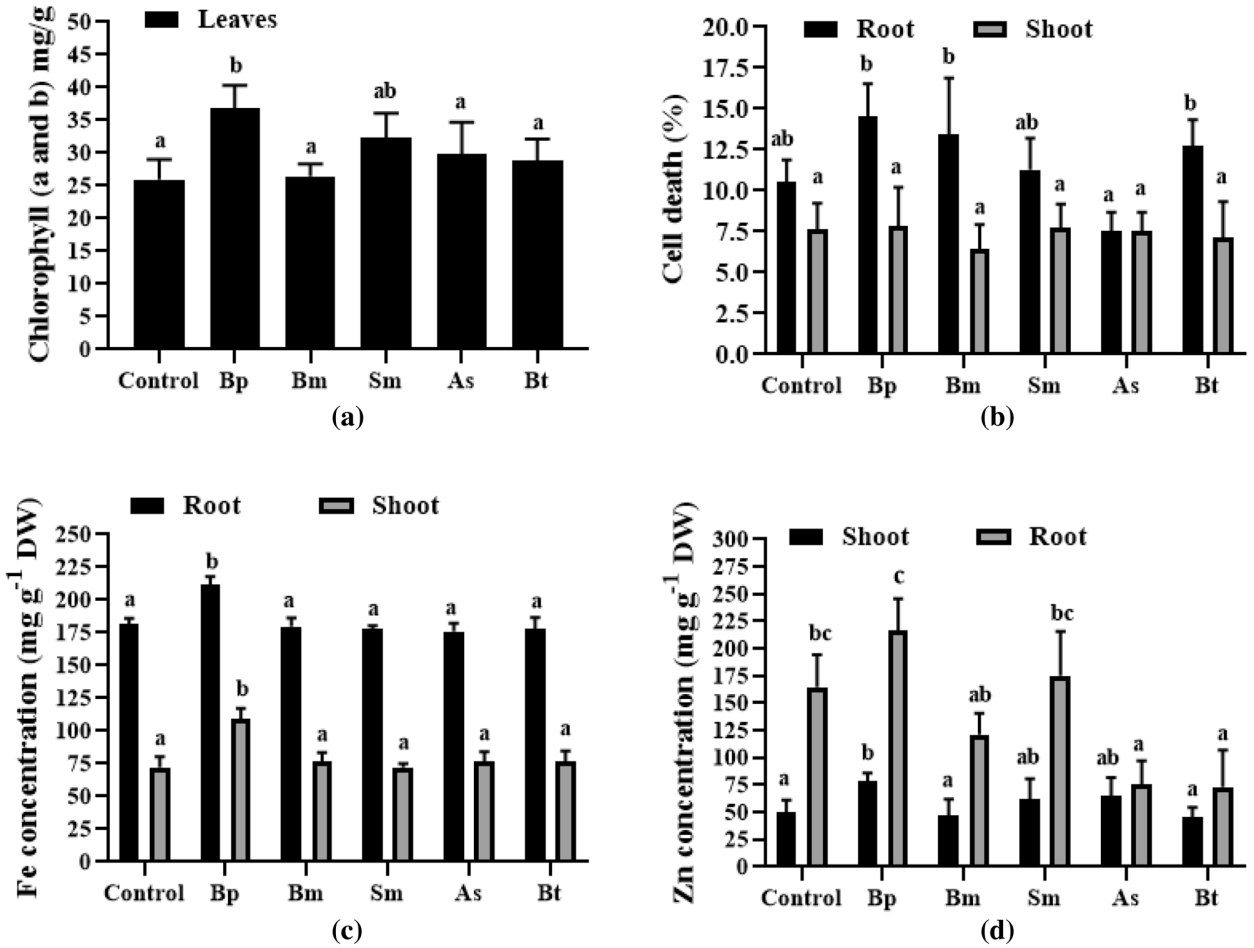
Chlorophyll, cell death, and Fe-Zn concentration of wheat plant treated with five different bacterial strains. Here, (**a**) indicates chlorophyll content in leaves, (**b**), (**c**), and (**d**) indicate cell death percentage, Fe concentration, Zn concentration, respectively, in both root and shoot. Different letters indicate significant differences between mean ± SD of replications (n = 3) at a *P* < 0.05 significance level.

All treatments had different chlorophyll concentrations (chlorophyll a and b) (i.e., Bp: 36.83 mg/g and control: 25.98 mg/g) (Fig. 4a). As a result, the Bp treatment likely had a higher photosynthetic capacity than the other inoculations. In the seeds that were treated with Bp, Fe and Zn concentrations were dramatically raised in both root and shoots (Fig. 4c,d), while in other stains, both Fe and Zn concentrations were identical with control plants (Fig. 4c,d). The percentage of cells that died is also shown in Fig. 4b.

### Antioxidant (APX, SOD, and CAT) analysis

**Figure 5:**
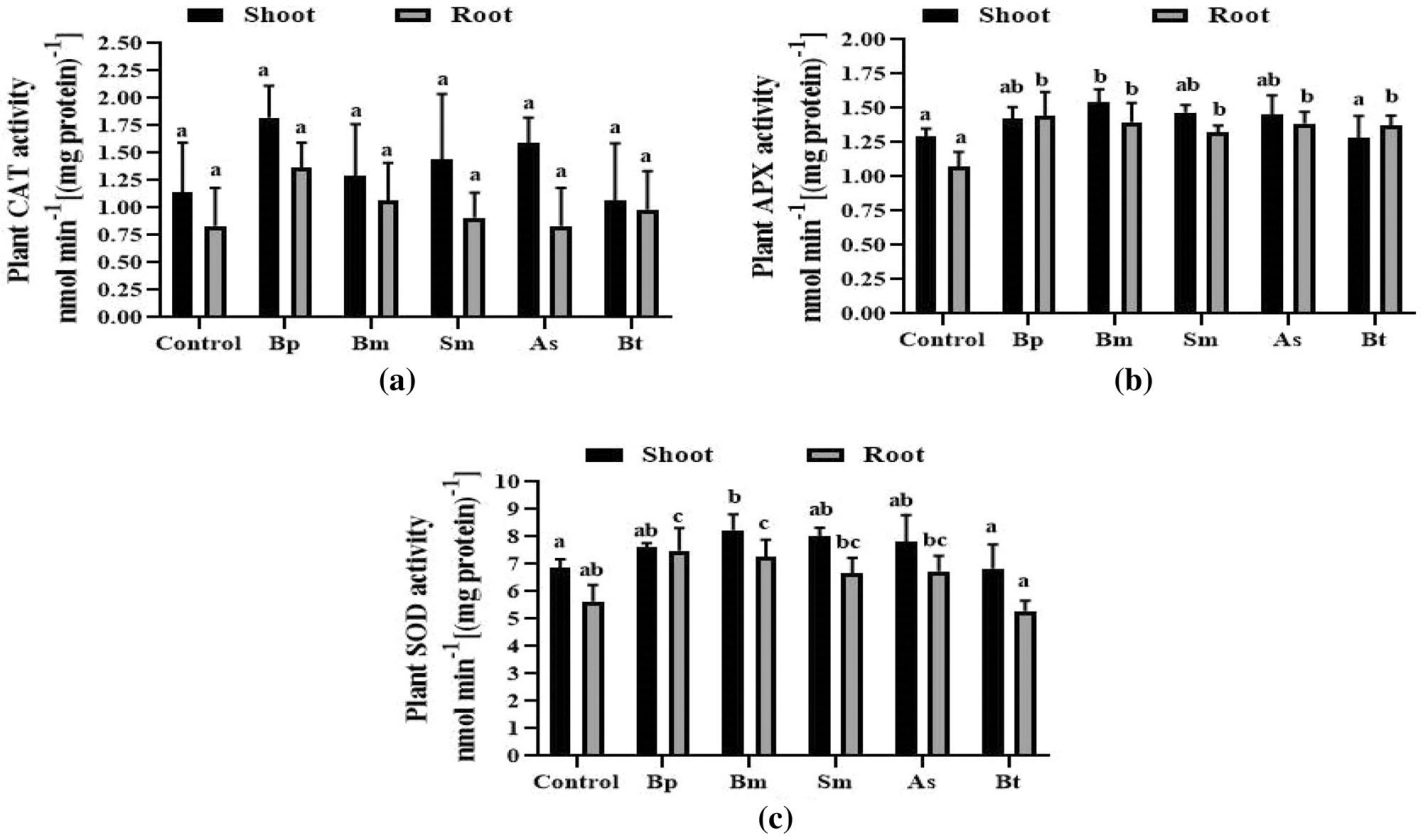
Antioxidant activity of wheat root and shoot treated with five different bacterial strains. Here, (**a**), (**b**), and (**c**) are indicating CAT, APX, and POD activity, respectively in both root and shoot. Different letters indicate significant differences between mean ± SD of replications (n = 3) at a *P* < 0.05 significance level.

Antioxidant activity was considerably higher in the Bp treatment but was only modestly increased in the other inoculations over the control. The highest levels of APX, SOD, and CAT activity were observed in the Bp inoculation (Fig. 5). As a result, this Bp inoculation had better antioxidant properties than other inoculations and controls.

### Screening of biofilm production

**Figure 6:**
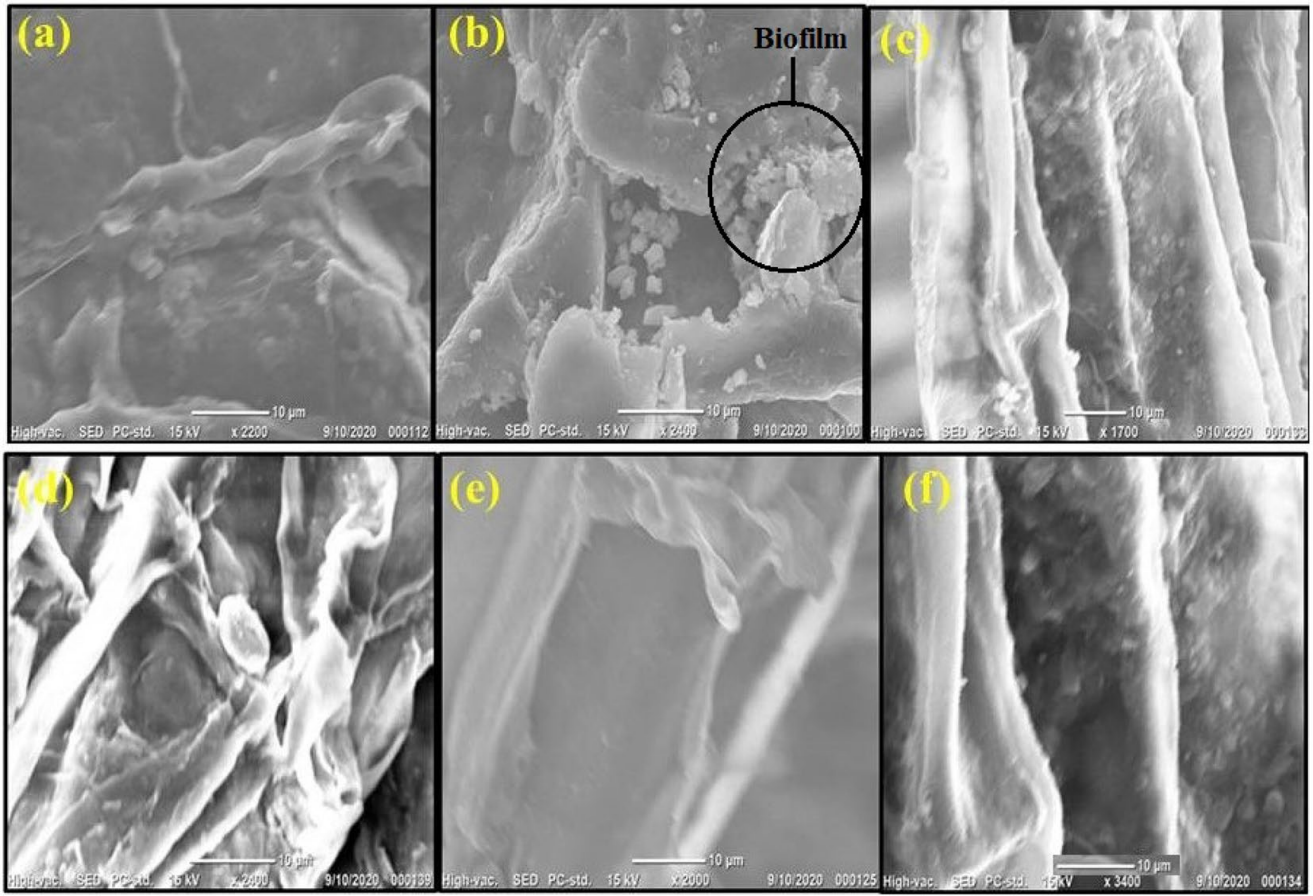
Screening Electron Microscopic observations visualizing biofilm in wheat root hairs treated with five different bacterial stains. Biofilms were observed after 15 days of drought stress induction. Here, (**a**), (**b**), (**c**), (**d**), (**e**), and (**f**) indicate control, Bp, Bm, Sm, As, and Bt inoculations, respectively.

The formation of biofilms is demonstrated in Fig. 6. In SEM screening, biofilm was formed in Bp inoculated plant's root hairs, but not in the control, Bm, Sm, As, and Bt inoculation plants' root hairs. Bp can form biofilms on wheat root hairs in drought conditions, which could assist plant survival.

### Identification of volatile compounds produced by *B. pseudomycoides*

**Figure 7:**
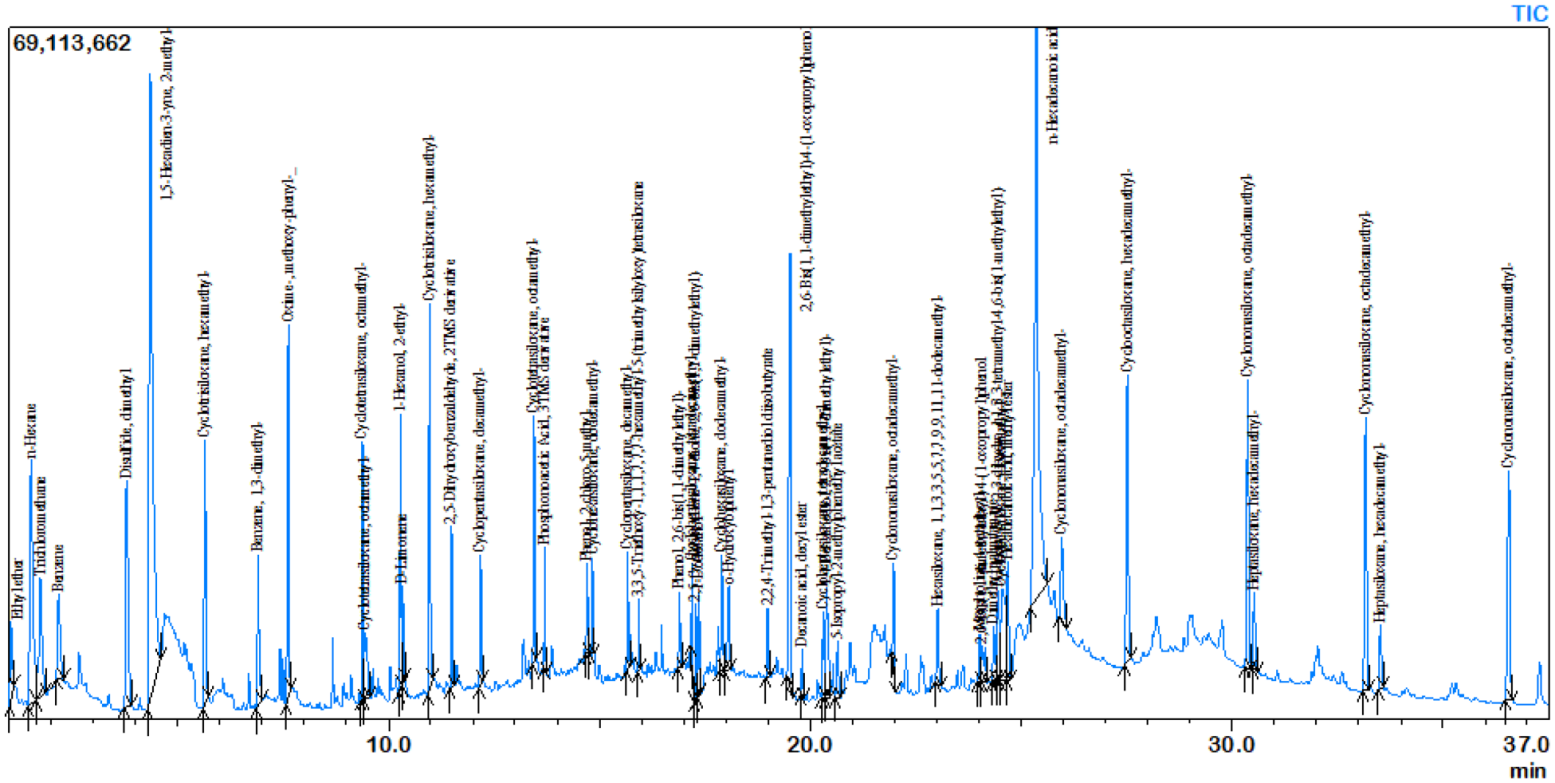
GC–MS chromatogram of volatile compounds produced by *B. pseudomycoides.*

For *B. pseudomycoides,* 45 volatile compounds were found in the GC–MS analysis. IUPAC name, PubChem CID, canolic smile, molecular weight and formula, retention time, area and height, and the GC–MS chromatogram are shown in Table S3 and Fig. 7.

### Observation of morphology and survival rate

In the pots, plant survival rates were 83.33%, 66.67%, 58.33%, and 16.67% for Bp, Bm, Bt, and Sm, respectively, however, neither the control nor As had any surviving plants after 15 days (Fig. S4 and Table S1). In the case of PEG treatment, Bp inoculation demonstrated the highest survival rates, 83.33%, where control was only 5% and other inoculations were less than 40% respectively, at 15% PEG treatment after artificially inducing drought; however other strains showed less drought tolerance than Bp (Table S2).

### Molecular expression

**Figure 8:**
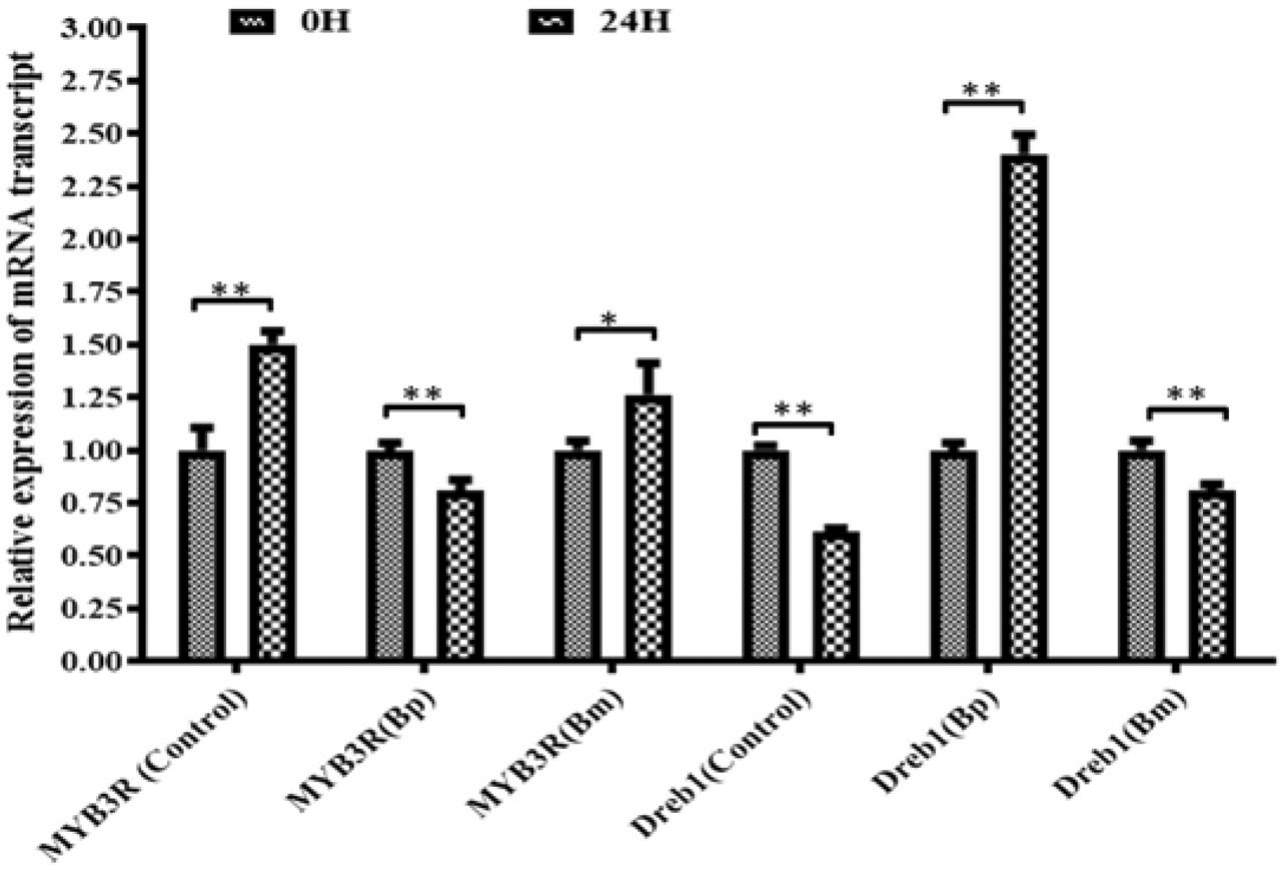
Quantitative analysis of expression of Dreb1 and MYB3R gene in leaves of wheat plants grown from seeds treated with Bp and Bm. Here, Bp and Bm indicate *B. pseudomycoides* and *B. massilioanrexius,* respectively. Data were recorded after 24 h of drought stress induction. Here, * indicates significance at the *P* = 0.05 level, and ** indicates significant at *P* = 0.01.

The extracted RNA showed two strong bands of 28S and 18S ribosomal RNA in 1% agarose gel and no significant RNA degradation was observed (Fig. S5). The Real-time PCR analysis showed that MYB3R and Dreb1 gene expression was comparatively similar in control, Bp, and Bm inoculation at 0-h drought induction. After 24 h post-induction, MYB3R gene expression was increased in the control and Bm inoculation but decreased in Bp inoculation, where Dreb1 gene was decreased in both control and Bm inoculation but increased in Bp inoculation (Fig. 8). The Dreb1 gene was expressed considerably more in the Bp inoculation than in the control but decreased in the Bm inoculation. In the case of Bp inoculation, Dreb1 gene was up regulated with the down-regulation of MYB3R gene.

### Homology modeling

**Figure 9:**
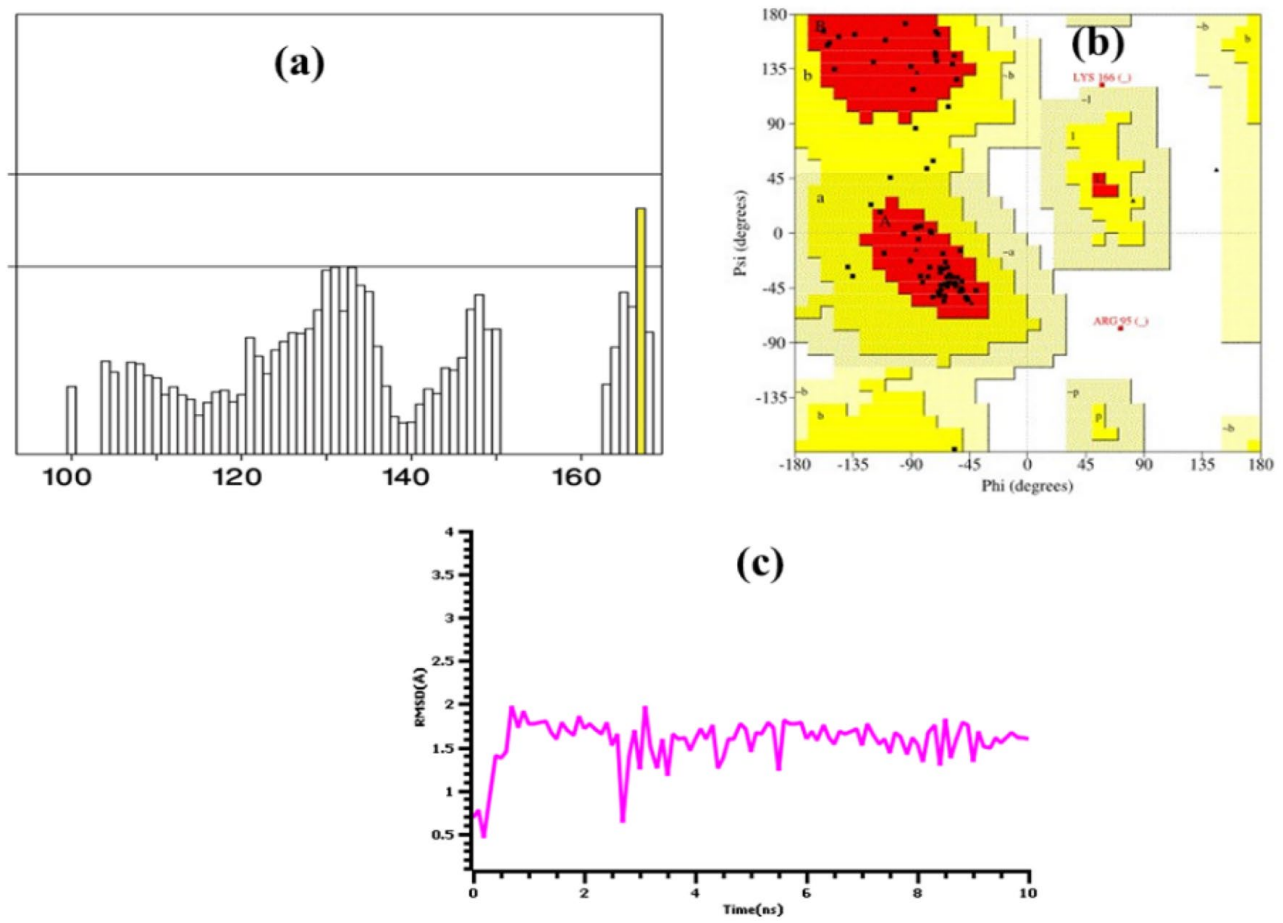
The quality assessments for the model protein, (**a**) ERRAT, where there were 85.55% residues in the allowed regions, (**b**) Ramachandran Plot assessment to validate structural quality, (**c**) root mean square deviation from the C-alpha atoms of the modeled protein, where lower deviations were observed.

Using Phyre2 tools, the MYB gene from *T. aestivum* was modeled and run for short dynamics for energy minimization processes. The structural stability and stiffness of the complex were investigated using the root mean square deviations of the C-alpha atoms from simulation trajectories over time. Because of their flexibility, the complexes exhibited a greater initial RMSD, as seen in Fig. 9c. However, after 2 ns, the MYB protein from wheat reached a steady state, and a lower degree of deviations was observed until the simulation's final periods. Ramachandran Plot analysis and ERRAT were used to further analyze the latest snapshots from the molecular dynamics simulations. The MYB protein from wheat had 85.55% residues in the allowed regions, 11.8% residues in the additional allowed regions, and 2.6% residues in the prohibited regions, as seen in Fig. 9a. A high-quality model structure is defined as a modeled protein with 90% of residues in the acceptable regions in Ramachandran plot assessments. The quality of the homology-modeled structure was also determined to be 98.113 (Fig. 9b) via ERRAT, where a good model structure has an ERRAT score of 80. Finally, the structures were used to perform molecular docking and simulations on the docked complex, as described below.

### Molecular docking study

**Figure 10:**
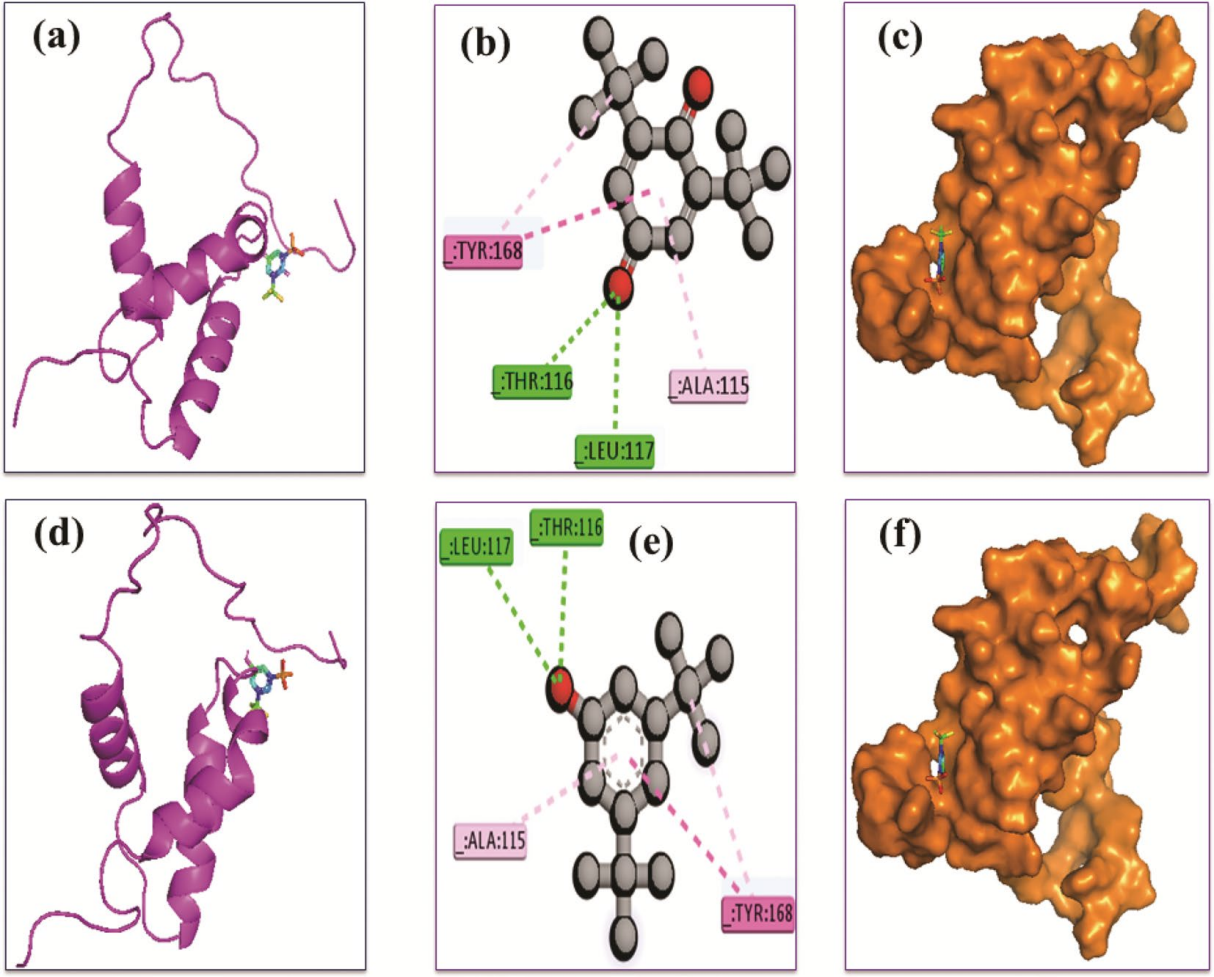
Docking simulation between MYB gene and 2,6-ditert-butylcyclohexa-2,5-diene-1,4-dione and 3,5-ditert-butylphenol, respectively, as a (**a**) cartoon view, (**b**) 3D view, (**c**) surface view of CID: 12,867 where (**d**) is a cartoon view, (**e**) 3D view, (**f**) surface view of CID: 70,825.

Two volatile compounds from *B. pseudomycoides* were investigated in this study based on their lower binding energies. These ligand molecules, 2,6-ditert-butylcyclohexa-2,5-diene-1,4-dione and 3,5-ditert-butylphenol, exhibited a higher binding affinity than other compounds. The binding energies of these compounds were −6.2 and −6.5 kcal/mol, respectively, indicating that 3,5-ditert-butylphenol and the MYB protein complex had higher binding energies than other compounds. The cartoon view, 3D view, and the surface view of these docking complexes are shown in Fig. 10a–f. Two hydrogen, two pi-alkyl, and one pi-sulfur bond was observed at Thr116, Leu117, Tyr168, Ala115, and Tyr168, respectively, stabilizing the 2,6-ditert-butylcyclohexa-2,5-diene-1,4-dione and MYB protein complex (Table 1 and Fig. 10b). Two hydrogen, two pi-alkyl, and one pi-sulfur bond was formed at Thr116, Leu117, Tyr168, Ala115, and Tyr168, respectively, in the 3,5-ditert-butylphenol and MYB protein complex (Table 1 and Fig. 10e).

**Table 1:** The docking interactions of MYB gene and the top two ligand molecules in molecular docking. The interactions were found using Discovery Studio software, where A, PA, PS H, indicate the alkyl, pi-alkyl, pi-sulfur, and hydrogen bond, respectively.

Complex	Amino acid residues	Bond type	Distance (Å)	Docking energy (Kcal/mol)
MYB protein and 2,6-ditert-butylcyclohexa-2,5-diene-1,4-dione	Thr116	H	2.24	−6.2
	Leu117	H	2.25	
	Tyr168	PS	4.97	
	Tyr168	PA	5.49	
	Ala115	PA	3.92	
MYB protein and 3,5-ditert-butylphenol	Thr116	H	2.03	−6.5
	Leu117	H	2.80	
	Tyr168	PS	5.03	
	Tyr168	PA	5.25	
	Ala115	PA	3.96	

### Molecular dynamics

**Figure 11:**
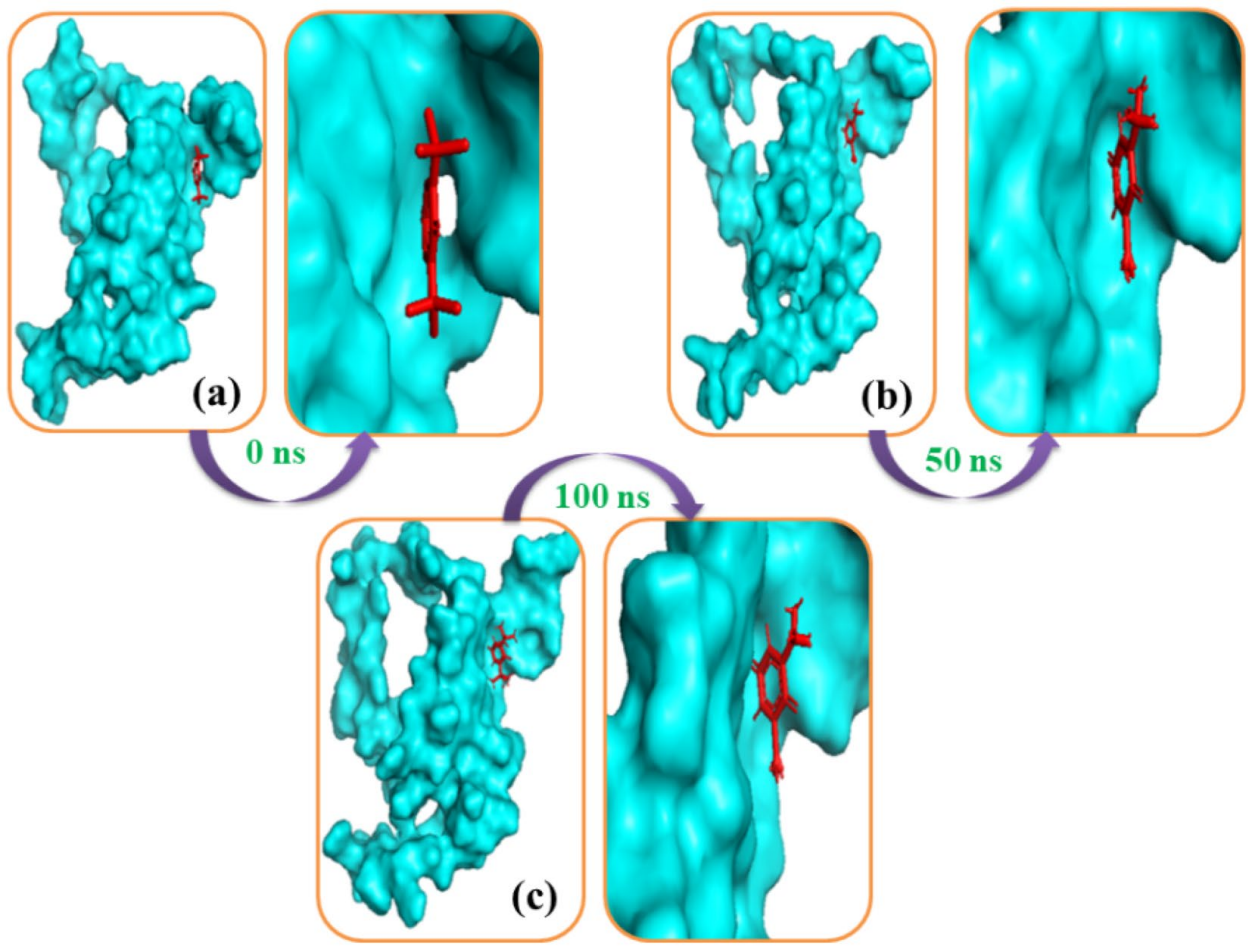
The simulation snapshots of MYB gene and 2,6-ditert-butylcyclohexa-2,5-diene-1,4-dione complexes acquired from the trajectories, where rigid profiles of the ligand–protein complex were observed in the same binding pockets. The snapshots (**a**–**c**) were taken after 0, 50, and 100 ns intervals, respectively.

**Figure 12:**
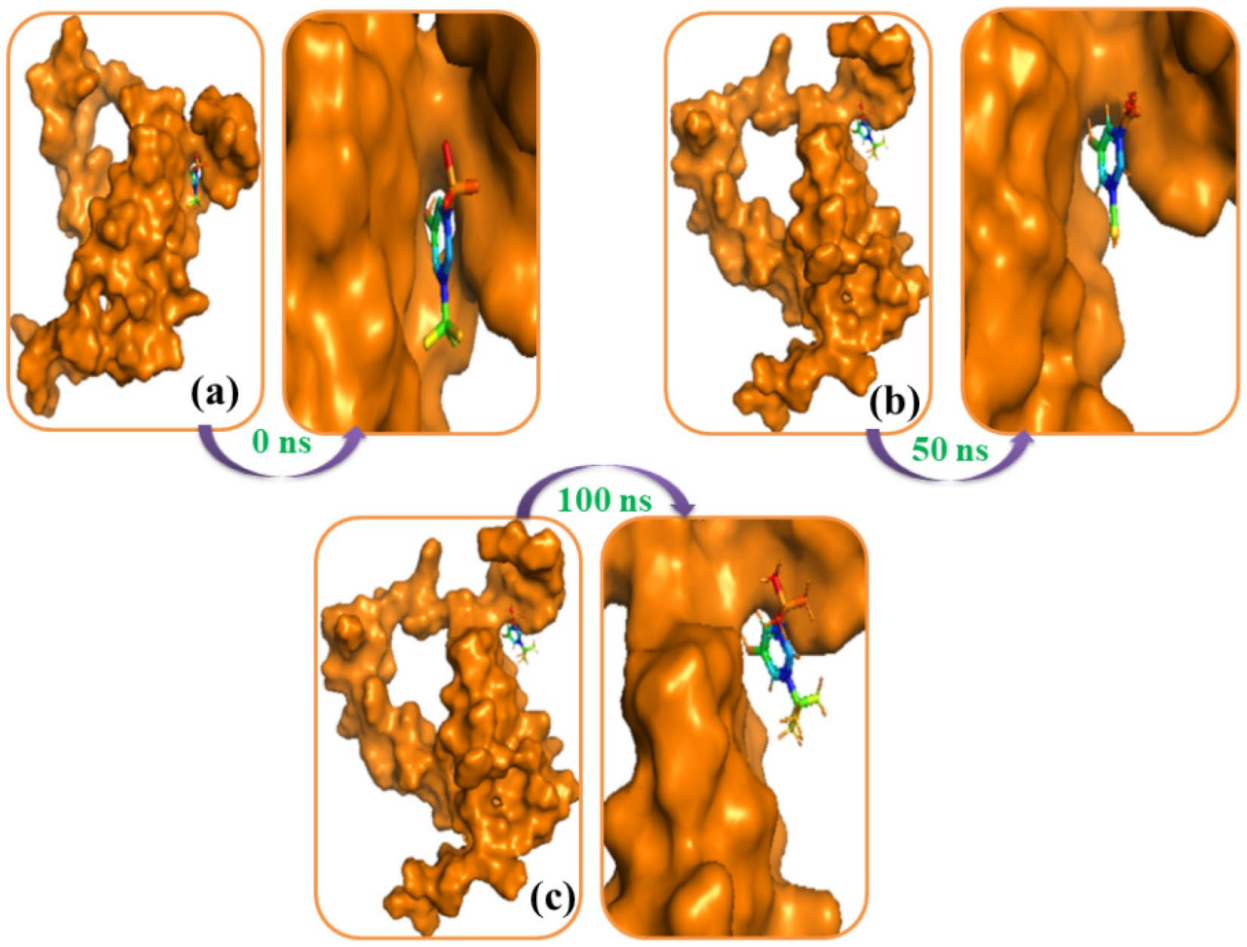
The simulation snapshots of MYB and 3,5-ditert-butylphenol complexes acquired from the trajectories, where rigid profiles of the ligand–protein complex were observed in the same binding pockets. The snapshots (a–c) were taken after 0, 50, and 100 ns intervals, respectively.

The goal of the molecular dynamics simulation was to understand the stable profile of docked complexes as well as the flexible behavior of ligand–protein systems. The 2,6-ditert-butylcyclohexa-2,5-diene-1,4-dione, and 3,5-ditert-butylphenol complexes showed reduced degrees of fluctuations over the simulation trajectories and hence demonstrated a stable mode during the simulation duration, as shown in Figs. 11 and 12. Furthermore, the complexes did not exceed 2.5 during the simulated timeframes, indicating that they are stable. Furthermore, the solvent-accessible surface area (SASA) of the simulated complexes was investigated to better understand the changes in the protein surface area, with a larger SASA indicating surface extension and a lower SASA indicating the truncated nature of the protein.

**Figure 13:**
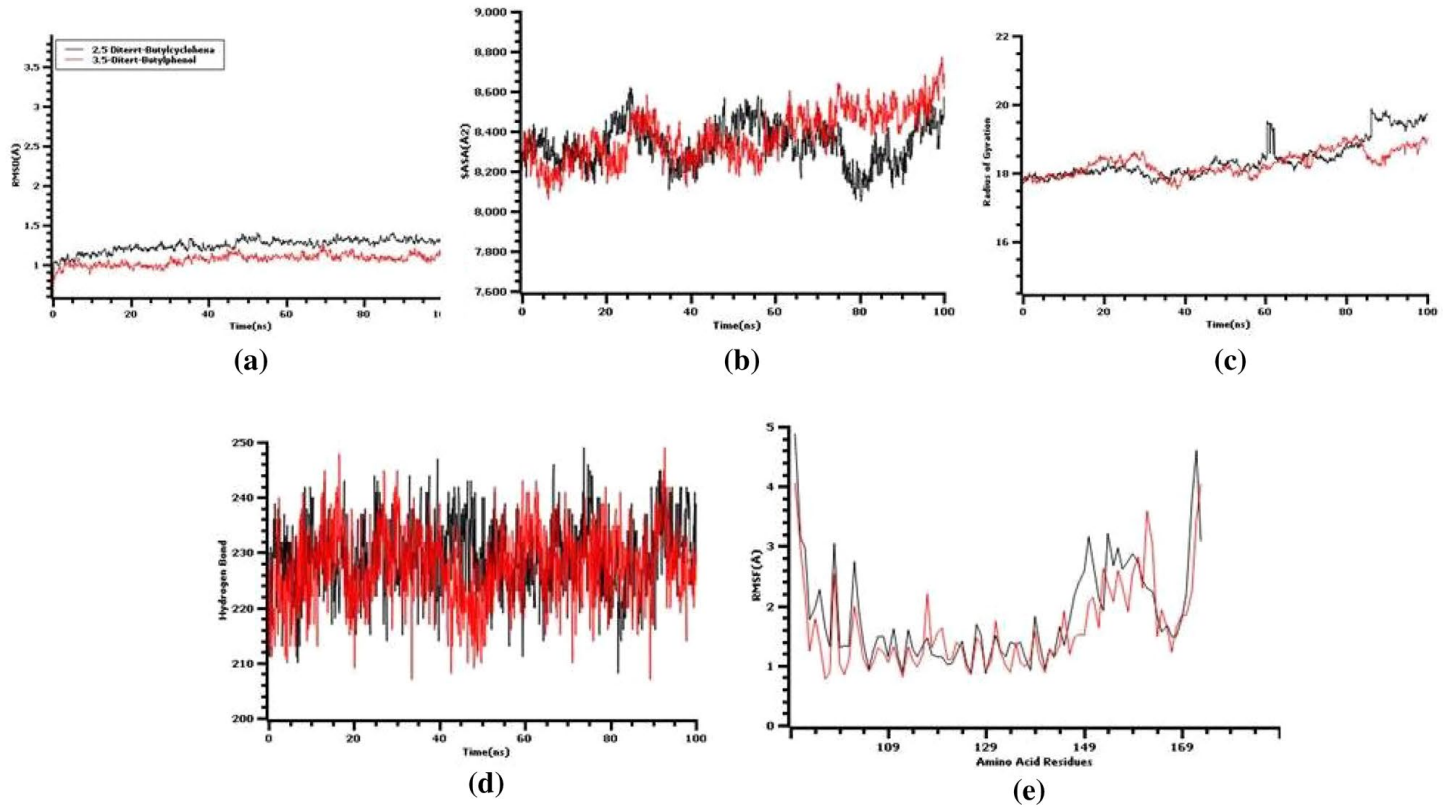
The molecular dynamics simulation. (**a**) Root mean square deviation of the two docked complexes, (**b**) solvent accessible surface area, (**c**) radius of gyration, (**d**) hydrogen bond of the docked complexes, and (**e**) root mean square fluctuation.

The lesser degree of the complexes' packing systems may be due to increased deviations at the initial points of the complexes with MYB and 2, 6-ditert-butylcyclohexa-2,5-diene-1,4-dione and 3,5-ditert-butylphenol, as seen in Fig. 13. As a result, after 70 ns, the SASA profiles of 2,6-ditert-butylcyclohexa-2,5-diene-1,4-dione complexes were reduced, probably due to the compacted shape of the ligand interaction with the protein systems. The degree of flexibility of the complexes is related to the radius gyration profile, with larger Rg descriptor deviations indicating a dynamic nature and lower Rg descriptor deviations indicating stable and stiff conformations. The 2, 6-ditert-butylcyclohexa-2, 5-diene-1,4-dione complexes showed changes at numerous sites between 50 and 60 ns, indicating that they are more mobile. The simulated systems' hydrogen bond patterning remained steady along the simulation trajectories, indicating that the complexes were stable. The root means square fluctuations of the simulated complexes also characterize the variations in amino acid residues in the simulating circumstances. According to Fig. 13, the complexes' maximal residues had a lower RMSF of less than 2.5, indicating that they were stable.

## Discussion

Global population expansions and global climate change are both concerns that impact cultivated crops. As a result, agricultural crops that can survive abiotic stresses are required around the world. Drought is a major abiotic phenomenon that reduces plant development and growth, affecting agricultural demands. Although many scientists believe that artificial gene transfer can be used to solve abiotic problems, there are many concerns regarding this methodology. PGPR, on the other hand, can spontaneously transmit genes. As a result, many scientists are attempting to use PGPR to further adapt drought-tolerant crop varieties. While this is not a novel method, researchers have recently applied this technique to the global climate change dilemma under abiotic stress circumstances. PGPR also reduces the use of chemical fertilizer by engaging in growth simulation. *T. aestivum* seeds germinate and grow faster after receiving beneficial bacterial inoculation^27^.

In our experiment, *B. pseudomycoides* inoculation was more effective than *B. massilioanrexius, S. marcescens, B. turingiensis,* and *Acinetobacter* sp. in terms of morphological growth, as measured by germination rate, potential, vigor index, potential, root-shoot length and number, and dry weight. Due to bacterial inoculation, changes in biochemical levels, functions, and morphology of *T. aestivum* could be observed. The substantial functional differences between the bacterial inoculation and control seeds were obvious throughout the entire experiment.

In the current study, we found that plants grown from *B. pseudomycoides-*inoculated seeds accumulated considerably more dry weight, RWC, and chlorophyll content. Similar agronomic improvements have been reported in the past with several bacterial inoculations^28–30^. Chlorophyll, a component of photosynthesis, has been shown to contribute to sorghum yield (in drought conditions) or improve sorghum yield and transpiration efficiency (in water-limited conditions)^31–33^. It has been observed that applying N_2_ to the seed surface improves germination, which is consistent with our findings. *B. pseudomycoides* inoculation was found to be more effective in boosting germination than other strains, showing that *B. pseudomycoides* could be an efficient in seed germination promoter. *B. pseudomycoides* inoculation in seeds resulted in an increase in total soluble protein and sugar in the root and shoot, demonstrating that *B. pseudomycoides* is effective in boosting cell metabolism and hence wheat plant growth and development. This observation is in line with prior wheat findings^34^. However, NO is an important signaling molecule that is involved in plant germination, growth, pollen tube growth, root organogenesis, flowering, physiology, biotic, abiotic, and developmental processes^35–39^, and in our study, NO levels were not significantly affected in the root but significantly increased after *B. pseudomycoides* inoculation.

In drought conditions, plant cells develop functional reactive oxygen species (ROS), whereas antioxidant enzymes inhibit ROS generation and reactivity to environmental stresses. Antioxidant enzymes such as SOD, CAT, and APX play a key role in reducing oxidative damage and ROS generation in plants, with CAT being the most essential in H_2_O_2_ scavenging to prevent ROS formation^41^. According to our findings, CAT, SOD, and APX activities increased in plants treated with *B. pseudomycoides*, but were lower in other bacterial treatments and non-treated plants. Iron uptake by electrons is reduced when Fe 3 + is reduced to Fe 2 + at the cell surface^40^. We also noticed that when *B. pseudomycoides* was inoculated, the concentrations of Fe and Zn in the root and shoot increased dramatically. Taran, Nataliya, et al. 2017 showed that Cu, Zn-nanoparticles reduced the detrimental effects of drought in wheat^41^. According to some studies, an adequate supply of Zn can modulate drought tolerance in crops like wheat, sunflower, tomato, and red cabbage^42–44^. So, inoculation with *B. pseudomycoides* may also affect electron discharge, facilitating iron uptake in wheat plants.

Under drought conditions, bacterial biofilm formation in plant root hairs is significant because it prevents the dispersion of bacterial biologically active substance secretion, the products of which then accumulate on the surface of the root to help plants absorb nutrients^45^. This biofilm formation encourages root-to-soil contact, which allows plants to absorb more nutrients from the soil. In response to certain PGPR strains, wheat produces hygroscopic polysaccharide-type alginates that improve water status, while the bacterial alginates' water-holding capacity slowly depletes^46–48^. This cellular metabolic adjustment caused by *B. pseudomycoides* wheat treatment adds to biofilm development, which reduces water loss. Flagella are required for the biofilm community to approach and travel across the surface, according to the widely accepted theory of biofilm formation, which holds that environmental cues start the process. Pili or LPSs, which are components of the outer membrane, mediate the initial stages of attachment. The development of quorum-sensing signals is conducive to the formation of a mature biofilm after the formation of micro colonies^49^. Surfactin was created during *B. subtilis* colonization^50^. This Surfactin contributes to the development of biofilms in plant root hair. It is known that Surfactin and other lipopeptides generated by *Bacillus* spp. cause plants to develop systemic resistance. As a result, *B. pseudomycoides’* alginate pathway may be responsible for drought tolerance. According to a recent study, several genes, including DREB1^51^, AtWRKY30^52^, TaASR1^53^, and TaMYB30-B^54^, considerably improve wheat drought stress tolerance. We chose DREB1 and MYB3R-1 genes expression after induced drought to illustrate wheat drought stress-related genes expression. *B. pseudomycoides* has a significant ability to naturally express the DREB1 gene but a lessened ability to express the MYB3R gene.

VOC released by PGPR bacteria have been shown to boost plant growth^55^, induce biofilm, and improve disease resistance^56^, iron nutrition, and abiotic stress tolerance^19,20^. For instance, *Pseudomonas chlororaphis* also produces 2,3-butanediol, a VOC that induces drought stress^57^. Homology modeling of myb protein and volatile compounds were used for molecular docking to discover the best-binding compounds to active regions of the targeted protein, MYB. Overexpression of myb reduces drought tolerance in transgenic *Arabidopsis*, as demonstrated by a decreased germination rate, shorter root length, lower proline content, and impaired POD, SOD, and CAT activities. Silencing myb expression in wheat enhanced antioxidant enzyme activity, proline content, and RWC in addition to activating DREB1 and DREB31 stress-related genes. The myb gene was found to have a negative effect on drought tolerance genes^58^. Here, the VOC 2, 6-ditert-butylcyclohexa-2, 5-diene-1, 4-dione, and 3, 5-ditert-butylphenol were shown to suppress MYB gene expression in wheat, possibly enhancing drought stress.

Wheat seeds inoculated with five distinct bacterial strains, including *B. pseudomycoides*, showed significant morphological improvement and chlorophyll content, leading to increased photosynthesis under normal and drought conditions. This inoculation also revealed drought morphological adaptation, PEG treatment, biofilm formation, and Dreb1 gene expression under drought conditions. *B. pseudomycoides* may also secrete several volatile compounds, with the best results coming from in silico study on 2, 6-ditert-butylcyclohexa-2, 5-diene-1, 4-dione, and 3, 5-ditert-butylphenol. Binding stability was demonstrated in a molecular simulation of protein and two volatile compounds.

## Methods

### Sample collection

Seed samples of *T. aestivum* (BARI gom-33) were collected from the Regional Wheat Research Center (Bangladesh Agricultural Research Institute, Shyampur Rajshahi-6212) and transported to the Microbiology Laboratory, Department of Genetic Engineering and Biotechnology, University of Rajshahi, Rajshahi, Bangladesh, for further analysis. Samples were treated with bacterial strains *B. pseudomycoides* (Bp)*, B. massilioanrexius* (Bm)*, B. thuringiensis* (Bt), *Serratia marcescens* (Sm), and *Acinetobacter* sp. (As), which were collected from this laboratory. We confirmed that all experiments related to plants (Seed samples of *T. aestivum* (BARI gom-33)) were in accordance with the relevant guidelines and regulations.

### Seed inoculation

Seeds were inoculated with freshly cultured bacterium stains according to the method described by Paul, et al. 2020^27^. Briefly, seeds were soaked and washed with distilled water for 20 min and then were sterilized with 70% (v/v) ethanol for 2.5 min. After washing thoroughly with distilled water, seeds were placed in a conical flask and inoculated with 100 mL of concentrated fresh bacterial culture. Seeds treated with the cultured medium alone were used as a control. After this, the conical flasks were placed on an orbital shaker and shaken for 6 h at ambient temperature at 160 rpm.

### Morpho-physiological characteristics

#### Germination parameters and plant growth

Germination parameters were screened following the method described by Zhou et al. 2016^59^. After treatment, 30 seeds were placed in 90 mm petri dishes that had four layers of wet tissue papers at the bottom, and the dishes were placed in a growth chamber set at 23 ± 2 °C and 50% humidity to monitor germination. After germination, the G_R_ (Germination Rate), G_P_ (Germination potential), I_G_ (Index of Germination), and L_I_ (Length index) were also calculated by the above reported method. After 10 days in petri dishes, plant growth was measured. Plants were carefully removed from the tissue paper, and the roots were cleaned with tap water. The lengths of the roots and shoots were measured and the number of roots tallied.

#### Root-shoot dry weights, ratio, and relative water content

The root and shoot dry weight measurements were carried out according to Arshadullar and Zaidi 2007^60^. The dry ratio was determined using the methods of Xu et al., 2015^61^. Five plants were randomly selected from each replication and rinsed with tap water to measure both shoot and root dry weights. Then shoots were then cut at the base and dried for 4 days at 70 °C in an incubator. The sample weights were determined using an electronic scale, and RWC (Relative Water Content) was measured according to^62^.

### Biochemical characterization

#### Chlorophyll (a and b) determination

The total chlorophyll content in leaves was measured according to Lichtenthaler and Wellburn 1983^63^. In this case, young fresh leaves were weighed and ground using a mortar and pestle in 90% methanol. Following grinding, the sample mixtures were centrifuged at 12,000 rpm for 10 min and the supernatants transferred. Finally, the optical density was recorded at 662 nm for chlorophyll a and 653 nm for chlorophyll b using a spectrophotometer (Analytic Gena, Germany), and the total chlorophyll content was calculated as mg/g.

#### Analysis of antioxidant enzymes (CAT, SOD, POD, and APX)

CAT, SOD, POD, and APX activities were measured according to Verma and Dubey, 2003^64^, Sun and Zigman, 1978^65^, Yordanova et al. 1999^66^, and Verma and Dubey, 2003^64^, respectively. In the case of all antioxidant activities, 0.3 g leaf and roots samples were crushed separately in phosphate buffer (10 mL, 100 mM, and pH 7.0). Then, the homogenate samples were centrifuged at 12,000 rpm for 12 min to separate the supernatant. For CAT activity, 2 mL of reaction mixtures containing potassium phosphate buffer (1.5 ml, 100 mM, pH 7.0), H_2_O_2_ (400 μl of 6%), and 100 μl leaf extract were prepared. Then, the decrease in absorbance was recorded at 240 nm by using a UV–Vis spectrophotometer (Analytic Gena, Germany). For SOD, reaction components were prepared by mixing sodium bicarbonate or carbonate buffer (1.3 mL, 50 mM, pH 9.8), EDTA (100 µl, 0.1 mM), and Epinephrine (500 µl, 0.6 mM). Then, adrenochrome formation was measured at 475 nm (Analytic Gena, Germany). In the case of APX, reaction mixtures were prepared by using potassium phosphate buffer (1 mL of 100 mM, pH 7.0), ascorbic acid (500 μl of 0.2 mM), EDTA (100 μl of 0.2 mM), H_2_O_2_ (300 μl of 6%), and 100 μl of plant extract, and the decrease of absorbance was recorded at 290 nm using a UV–VIS spectrophotometer (Analytic Gena, Germany) at 10 s intervals up to 1 min. The specific activity of the enzyme was expressed as μmol min^-1^ (mg protein)^-1^.

#### Determination of Fe and Zn concentration

The concentrations of Zn and Fe were determined according to Kabir et al. 2017^67^. For this, fresh plant tissues were washed with CaSO_4_ (1 mM) for 5 min and then thoroughly washed with distilled water. After this, clean samples were dried in an incubator at 80 °C for 6 days. A total of 3 mL of HNO_3_ and 1 mL of H_2_O_2_ were added to each sample in the test tube and heated at 75 °C for 12 min. Zn and Fe concentrations were evaluated by Flame Atomic Absorption Spectroscopy, using a ASC-6100 auto sampler and air-acetylene atomization gas mixture system (Model No.AA-6800, Shimadzu), where standard solutions of Zn and Fe were separately prepared from their respective concentrations.

#### Determination of H_2_O_2_, cell death, and NO

H_2_O_2_ was measured according to Alexieva et al. 2001^68^, cell death was determined according to Jacyn Baker and Mock, 1994^69^, and NO was estimated according to Orozco-Cárdenas et al., 2002^70^. In case of H_2_O_2_, fresh plant samples were separately homogenized in 0.1% Trichloroacetic acid (TCA) and centrifuged at 10,000 rpm for 14 min. The supernatants were mixed with KI (1 M) and phosphate buffer (10 mM, pH 7.0) then kept in the dark for 1 h, and the optical density (OD) of extracts were taken at 390 nm by spectrophotometer (Analytic Gena, Germany). For cell death determination, fresh samples were incubated at 25 °C in 0.25% Evans blue solution for 16 min, then in 1.0 mL of 80% ethyl alcohol for 10 min, and finally incubated in a water bath at 50 °C for 15 min. After this, the mixtures were centrifuged at 12,000 rpm for 10 min. Then, the absorbance was measured at 600 nm (Analytic Gena, Germany) and the amount of cell death calculated based on fresh weight. For NO determination, samples were homogenized in cooled NO buffer (1 mL) and centrifuged at 10,000 rpm for 10 min. The supernatants were transferred to a 5 mM oxyhemoglobin (HbO_2_) solution and incubated for 7 min at room temperature. Optical density was measured at 401 nm (Analytic Gena, Germany) to determine the conversion rate of oxyhemoglobin (HbO_2)_ to methemoglobin (metHb), which is an indicator of the presence of NO.

#### Estimation of soluble sugar and protein

The total soluble sugar content of the plant samples was determined using the method of M. Dubois, et al., 1956^71^, and soluble protein content was determined using the Bradford protein assay according to He, 2011^72^. For soluble sugar, the fresh samples were homogenized in 90 °C hot aqueous ethanol (v/v 80%) and centrifuged at 12,000 rpm for 10 min. Following centrifugation, the supernatants were mixed with 0.2% anthrone reagent and incubated in a boiling water bath for 10 min. After cooling on ice, the optical density OD was recorded at 620 nm (Analytic Gena, Germany). In the case of soluble protein, homogenized samples with 2 mM EDTA (Ethylenediamine Tetraacetic Acid), 50 mM Tris–HCl, pH 7.5 and 0.04% (v/v) 2-mercaptoethanol added were centrifuged at 12,000 rpm for 15 min at 25 °C. The supernatant was mixed with 1 mL CBB (Coomassie Brilliant Blue), and the optical density was determined at 595 nm using spectrophotometer (Analytic Gena, Germany).

#### Screening of volatile compounds

Volatile compounds were analyzed according to P. Fincheira et al., 2017^73^. For this, 20 mL of each bacterial culture was inoculated into 30 mL of LB liquid medium in a vial and incubated at 30 °C for 6 days with constant shaking. The SPME fiber was then inserted into the headspace of the vial and heated to 50 °C for 30 min. SPME fibers were desorbed at 220 °C for 5 min, and GC–MS was run for 25 min using GC–MS, Shimadzu, with helium gas as the carrier. The column temperature was set to 35 °C for 3 min before gradually increasing to 180 °C at a rate of 10 °C/min. After this, the temperature was increased to 240 °C for 5 min, and the mass spectrometer was run in electron ionization mode at 70 Ev with a temperature of 220 °C and a continuous scan from 50 to 500 m/z. The results were compared to the Mass Spectrum Library of the NIST/EPA/NIH.

### Drought stress tolerance activity

#### Morphological stress observation

For drought stress analysis, eight plants were selected in each treatment with three replications, and water was discontinued for 15 days to assess vulnerability in pot conditions. After stress induction, discussed below, the plants were watered and given four days to recover. The plants that were rescued were counted as survivors.

#### PEG treatment

PEG (MW: 6000) was used to induce drought stress in wheat, with three treatments: control (0% PEG), moderate drought stress (10% PEG), and severe drought stress (15% PEG). Every day, each petri dish received a total of 10 doses of 350 mL PEG solution. The drought stress period lasted 12 days for each treatment.

#### Screening of biofilm formation

After 15 days of artificial drought stress, plants were removed from the soil, and roots were safely rinsed with tap water to remove adhering dirt. The roots were then split with scissors and placed on a carbon tape before being coated by gold using a Sputter Coater 108 auto Cressington. Finally, the samples were examined via a scanning electron microscope (JCM-6000 Plus (JEOL Japan) at various wavelengths for scanning biofilm.

#### RNA isolation and gene expression analysis

RNA isolation and gene expression were analyzed by following the method described by Rahman et al. 2018^74^. Briefly, two genes, Dreb1 and MYB3R, were chosen for gene expression analysis in leaves using quantitative reverse transcription PCR techniques. A total of 30 mg of leaves were ground in liquid nitrogen using a mortar and pestle, and total RNA was extracted using Promega SV total RNA isolation system. After DNase treatment, 1 μl extracted RNA of some samples was run in 1% agarose gel to observe the level of RNA degradation. First-strand cDNA was produced using Promega, Reverse Transcription systems, and the RNA extraction was confirmed by MoF buffer in agarose gel electrophoresis and quantified by Nano drop. After this, RNase was used to clean the cDNA, and real-time PCR analysis was performed using an Eco™ real-time PCR system controlled by Eco Software v 4.7.0 (Illumina. USA), with a program of 3 min at 95 °C, 40 cycles of 30 s at 94 °C, 15 s at 56 °C and 30 s at 72 °C. Expression was normalized using actin as an internal control.

#### Homology modeling

The crystal structure of the MYB-like domain-containing protein of *T. aestivum* had not yet explored in the protein database (RCSBD). So, for further protein modeling and molecular docking study, we used sequences from the UniPort database, MYB (UniProt ID: A0A3B6RJY9). The protein sequences’ fasta format was extracted and entered into the Phyre2 web tools. The C chain of a specific DNA complex of the MYB DNA-2 binding domain with cooperative recognition helices (PDB ID: 1MSE) was chosen as a template, with confidence and percent identity of 100, and 56 for the MYB gene, respectively. The MYB model protein was exposed to short molecular dynamics simulations (10 ns) for refinements and minimizations. Further validations were performed using the last snapshots from the simulation trajectories. ERRAT and Ramachandran Plot analysis was used to further assess the reduced structures.

#### Ligand preparation

The volatile compounds of *B. pseudomycoides* were derived through GC–MS analysis, and the sdf format of 3D structures was retrieved from the PubChem database (http://www.pubchem.ncbi.nlm.nih.gov/)^75^. The mmff94 force field was used to construct and optimize the structure of the ligands with the aid of Avogadro software^76^.

#### Molecular docking

Molecular docking is an important tool for identifying the best compounds to fit receptor molecules based on docking energy. The Auto Dock Vina software package was used for performing molecular docking. The ligands were converted to PDBQT format, and the grid box center was set to (X: 26.29 Å, Y:12.60 Å Z:58.94 Å) and (X:50.33 Å, Y:67.27 Å, Z:59.25 Å), respectively^77–79^. Docking was performed using the Lamarckian algorithm, with the parameters set to 250 runs and 25,000,000 energy assessments for each cycle. The level of thoroughness was set to 8. Finally, docking calculations were run in AutoDock Vina. The Discovery Studio (version 3.0)^80^, and Pymol software was used (version 2.3)^81^ for binding interactions analysis.

#### Molecular dynamics simulation

The molecular dynamics simulation was performed by the YASARA dynamics package using the AMBER14 force field^82,83^. The docked complexes were cleaned, optimized, and a hydrogen bond networking system was used with a pH of 7.4, a temperature of 310 K, and a 0.9% NaCl concentration. A TIP3P solvation model was used with periodic boundary conditions to produce a cubic simulations cell. Simulated annealing methods were used to do fundamental energy minimization in steepest-gradient algorithms (5000 cycles). Long-range electrostatic interactions were calculated by the Particle Mesh Ewalds (PME), with a cut-off radius of 8.0 Å. The simulation cell was extended by 20 Å in each case from the complexes. The simulations were run using a time step of 2.0 fs. The final simulation was run for 100 ns^84^, while adhering to constant pressure and a Berendsen thermostat. The root mean square deviation, solvent accessible surface area, radius of gyration, and hydrogen bonding were all calculated using simulated trajectories stored at 100 ps interval^85–92^.

### Statistical analysis

The experiment was performed using a CRBD (Complete Randomized Block Design) with three replication of each biological sample. The DMRT (Duncan’s Multiple Range Test) was used to analyze the significance of each group’s data at a *P* ≤ 0.05 level of significance in a one-way ANOVA in SPSS Statistics 26 software. Graph Pad Prism 8.0.2.263 was used for preparing all figures.

## Conclusion

Wheat growth is enhanced by seed inoculation with *B. pseudomycoides* in a normal environment, with increased of total soluble sugar, protein, chlorophyll concentrations, RWC, and root-shoot length. This inoculation can form biofilm in drought stress, which supports morphological drought stress and expresses drought response genes. Molecular evidence revealed that *B. pseudomycoides* has a strong ability to spontaneously express the DREB1 gene but a weak ability to express the MYB gene. The overexpression of MYB gene suppresses the expression of DREB1 gene. *B. pseudomycoides* secreted volatile compounds such as 2, 6-ditert-butyl cyclohex-2, 5-diene-1, 4-dione, and 3, 5-ditert-butylphenol, which had binding affinity and stability in molecular docking and simulation. *B. pseudomycoides*, as well as 2, 6-ditert-butyl cyclohex-2, 5-diene-1, 4-dione, and 3, 5-ditert-butylphenol, may enhance DREB1 gene expression by suppressing MYB gene under drought conditions. Microorganisms may be able to help mitigate the detrimental effects of climate change on crop growth, according to the study. However, more investigation into the subject is required, as well as clarification of the protective method.

## Supplementary Information

Supplementary Information.

## Data Availability

All data generated or analyzed during this study are included in this published article and its supplementary information files.

## Abbreviations

RWC: Relative water content
H_2_O_2_: Hydrogen peroxide
NO: Nitric oxide
CAT: Catalase
SOD: Superoxide dismutase
POD: Peroxidases
APX: Ascorbate peroxidase
RMSD: Root-mean-square deviation
SASA: Solvent accessible surface area
Rg: Radius of gyration
RMSF: Root mean square fluctuation
ROS: Reactive oxygen species
PGPR: Plant-growth promoting rhizobacteria
VOCs: Volatile compounds
SEM: Scanning electron microscopy
GC–MS: Gas chromatography–mass spectrometry
IUPAC: International union of pure and applied chemistry
PEG: Polyethylene glycol
G_R_: Germination rate
G_P_: Germination potential
I_G_: Index of germination
L_I_: Length index
EDTA: Ethylenediamine tetraacetic acid
HbO_2_: Oxyhemoglobin
metHb: Methemoglobin
CBB: Coomassie brilliant blue
SPME: Solid phase microextraction
cDNA: Complementary DNA
RNA: Ribonucleic acid
RNase: Ribonuclease
PCR: Polymerase chain reaction
YASARA: Yet another scientific artificial reality application
NaCl: Sodium chloride
PME: Particle mesh Ewalds
