# Supplementary material for: Volatile compounds of Bacillus pseudomycoides induce growth and drought tolerance in wheat (Triticum aestivum L.)
Source: Sci Rep. 2022 Nov 9;12:19137. doi: 10.1038/s41598-022-22354-2 (PMC9646913; doi:10.1038/s41598-022-22354-2)
Supplement: Supplementary file 1 — Supplementary Information. [file 41598_2022_22354_MOESM1_ESM.docx]

**Volatile compounds of *Bacillus pseudomycoides* induce growth and drought tolerance in wheat (*Triticum aestivum* L.)**

**Gobindo Kumar Paul^1,4^, Shafi Mahmud^2^, Amit Kumar Dutta^3^, Swagotom Sarkar^4^, Aysha Akter Laboni^4^, Md. Shamim Hossain^5^, Abir Nagata^6,7^, Pranab Karmaker^4^, Mamudul Hasan Razu^4^, Taheruzzaman Kazi^6^, Md. Salah Uddin^1^, Shahriar Zaman^1^, Md Sayeedul Islam^8^, Mala Khan^4,*^, Md. Abu Saleh^1,*^**

^1^Microbiology Laboratory, Department of Genetic Engineering and Biotechnology, University of Rajshahi, Rajshahi-6205, Bangladesh

^2^Division of Genome Sciences and Cancer, The John Curtin School of Medical Research, and The Shine-Dalgarno Centre for RNA Innovation, The Australian National University, Canberra, ACT 2601, Australia.

^3^Department of Microbiology, University of Rajshahi, Rajshahi-6205, Bangladesh

^4^Bangladesh Reference Institute for Chemical Measurements (BRiCM), Dhaka, Bangladesh

^5^Department of Biotechnology and Genetic Engineering, Islamic University, Kushtia-7003, Bangladesh

^6^Department of Regenerative Dermatology, Graduate School of Medicine, Osaka University, Suita 565-0871, Japan

^7^International Platform for Dryland Research and Education, Tottori University, Yonago 680-0001, Japan

^8^Department of Biological Sciences, Graduate School of Science, Osaka University, Machikaneyama-cho 1-1, Toyonaka, Osaka 560-0043, Japan

*Corresponding Author: Md. Abu Saleh ([saleh@ru.ac.bd](mailto:saleh@ru.ac.bd))

Cell No. +8801716731747

Fax No. +880721750064

Mala Khan (malakhan_07@yahoo.com)

**Table S1:** Plant surviving number and surviving percentage of wheat treated with different bacterial species. Data were recorded after 15 days of drought induction.

| **Samples Name** | **Surviving number of plants after days** | | | | | | **Survival rate after 15 days inducing drought (%)** |
| --- | --- | --- | --- | --- | --- | --- | --- |
|  | 0 days | 3 days | 6 days | 9 days | 12 days | 15 days |  |
| **Control** | 24 | 24 | 8 | 0 | 0 | 0 | 0 |
| **Bp** | 24 | 24 | 23 | 22 | 22 | 20 | 83.33 |
| **Bm** | 24 | 24 | 20 | 19 | 17 | 14 | 66.67 |
| **Sm** | 24 | 24 | 17 | 13 | 9 | 4 | 16.67 |
| **As** | 24 | 24 | 19 | 10 | 2 | 0 | 0 |
| **Bt** | 24 | 24 | 23 | 20 | 18 | 16 | 58.33 |

**Table S2:** The effect different percentage of PEG treatment on plant survival rate

| **Name of the samples** | **Effect of PEG on plant survival rate**  **(Mean**±**SD)** | | | |
| --- | --- | --- | --- | --- |
|  | **1 (%)** | **5 (%)** | **10 (%)** | **15 (%)** |
| **Control** | 95.00±5.00**^a^** | 78.33±7.64**^a^** | 46.67±5.77**^a^** | 5.00±5.00**^a^** |
| **Bp** | 100.00±0.0**^a^** | 98.33±2.89**^b^** | 96.67±2.89**^d^** | 83.33±5.77**^d^** |
| **Bm** | 100.00±0.0**^a^** | 88.33±5.77**^ab^** | 73.33±2.89**^c^** | 36.67±7.64**^c^** |
| **Sm** | 96.67±2.89**^a^** | 76.67±2.89**^a^** | 53.33±7.64**^ab^** | 20.00±5.00**^b^** |
| **As** | 96.67±5.77**^a^** | 83.33±7.64**^a^** | 58.33±12.58**^ab^** | 26.67±2.89**^b^** |
| **Bt** | 100.00±0.0**^a^** | 86.67±7.64**^a^** | 61.67±5.77**^bc^** | 38.33±2.89**^c^** |

**Table S**3**:** Volatile components identification of *Bacillus psudomycoides* by GC-MS.

| Compound Name | IUPAC Name | Canolic Smile | PubChem CID | Molecular Weight (g/mol)& Formula | % Area | Ret Time | % Height |
| --- | --- | --- | --- | --- | --- | --- | --- |
| Ethyl ether | ethoxyethane | CCOCC | 3283 | 74.12  [C_4_H_10_O](https://pubchem.ncbi.nlm.nih.gov/#query=C4H10O) or (C_2_H_5_)_2_O or CH_3_CH_2_OCH_2_CH_3_ | 0.96 | 1.043 | 0.96 |
| n-Hexane | Hexane | CCCCCC | 8058 | 86.18  [C_6_H_14_](https://pubchem.ncbi.nlm.nih.gov/#query=C6H14) | 4.82 | 1.043 | 3 |
| Trichloromethane | chloroform | C(Cl)(Cl)Cl | 6212 | 119.37  [CHCl_3_](https://pubchem.ncbi.nlm.nih.gov/#query=CHCl3) | 2.37 | 1.741 | 1.46 |
| Benzene | benzene | C1=CC=CC=C1 | 241 | 78.11   \| C_6_H_6_ \| \| --- \| | 1.25 | 2.175 | 1.04 |
| Disulfide, dimethyl | (methyldisulfanyl)methane | CSSC | 12232 | 94.2  [C_2_H_6_S_2_](https://pubchem.ncbi.nlm.nih.gov/#query=C2H6S2) | 3.41 | 3.787 | 2.77 |
| 1,5-Hexadien-3-yne, 2-methyl- | 2-methylhexa-1,5-dien-3-yne | CC(=C)CCC=C | 13176 | 92.14  [C_7_H_8_](https://pubchem.ncbi.nlm.nih.gov/#query=C7H8) | 13.01 | 4.351 | 7.65 |
| Cyclotrisiloxane, hexamethyl- | 2,2,4,4,6,6-hexamethyl-1,3,5,2,4,6-trioxatrisilinane | C[Si]1(O[Si](O[Si](O1)(C)C)(C)C)C | 10914 | 222.46 [C_6_H_18_O_3_Si_3_](https://pubchem.ncbi.nlm.nih.gov/#query=C6H18O3Si3) | 2.41 | 5.647 | 3.22 |
| Benzene, 1,3-dimethyl- | 1,3-xylene | CC1=CC(=CC=C1)C | 7929 | 106.16  [C_8_H_10_](https://pubchem.ncbi.nlm.nih.gov/#query=C8H10) or C_6_H_4_(CH_3_)_2_ | 1.72 | 6.897 | 1.85 |
| Oxime-, methoxy-phenyl-_ | methyl (*Z*)-*N*-hydroxybenzenecarboximidate | COC(=NO)C1=CC=CC=C1 | 9602988 | 151.16  [C_8_H_9_NO_2_](https://pubchem.ncbi.nlm.nih.gov/#query=C8H9NO2) | 3.51 | 7.62 | 4.57 |
| Cyclotetrasiloxane, octamethyl- | 2,2,4,4,6,6,8,8-octamethyl-1,3,5,7,2,4,6,8-tetraoxatetrasilocane | C[Si]1(O[Si](O[Si](O[Si](O1)(C)C)(C)C)(C)C)C | 11169 | 296.61  [C_8_H_24_O_4_Si_4_](https://pubchem.ncbi.nlm.nih.gov/#query=C8H24O4Si4) | 1.68 | 9.382 | 3.18 |
| Cyclotetrasiloxane, octamethyl- | 2,2,4,4,6,6,8,8-octamethyl-1,3,5,7,2,4,6,8-tetraoxatetrasilocane | C[Si]1(O[Si](O[Si](O[Si](O1)(C)C)(C)C)(C)C)C | 11169 | 296.61  [C_8_H_24_O_4_Si_4_](https://pubchem.ncbi.nlm.nih.gov/#query=C8H24O4Si4) | 1.22 | 9.442 | 0.81 |
| 1-Hexanol, 2-ethyl- | 2-ethylhexan-1-ol | CCCCC(CC)CO | 7720 | 130.23  [C_8_H_18_O](https://pubchem.ncbi.nlm.nih.gov/#query=C8H18O) or CH_3_(CH_2_)_3_CH(CH_2_CH_3_)CH_2_OH | 1.82 | 10.285 | 3.36 |
| D-Limonene | (4*R*)-1-methyl-4-prop-1-en-2-ylcyclohexene | CC1=CCC(CC1)C(=C)C | 440917 | 136.23  [C_10_H_16_](https://pubchem.ncbi.nlm.nih.gov/#query=C10H16) | 0.65 | 10.336 | 1.19 |
| Cyclotrisiloxane, hexamethyl- | 2,2,4,4,6,6-hexamethyl-1,3,5,2,4,6-trioxatrisilinane | C[Si]1(O[Si](O[Si](O1)(C)C)(C)C)C | 10914 | 222.46 | 3.04 | 10.979 | 4.72 |
| 2,5-Dihydroxybenzaldehyde, 2TMS derivative | 2,5-bis(trimethylsilyloxy)benzaldehyde | C[Si](C)(C)OC1=CC(=C(C=C1)O[Si](C)(C)C)C=O | 622536 | 282.48  [C_13_H_22_O_3_Si_2_](https://pubchem.ncbi.nlm.nih.gov/#query=C13H22O3Si2) | 1.14 | 11.486 | 1.98 |
| Cyclopentasiloxane, decamethyl- | 2,5-bis(trimethylsilyloxy)benzaldehyde | C[Si](C)(C)OC1=CC(=C(C=C1)O[Si](C)(C)C)C=O | \| 622536 \| \| --- \| | 282.48   \| [C_13_H_22_O_3_Si_2_](https://pubchem.ncbi.nlm.nih.gov/#query=C13H22O3Si2) \| \| --- \| | 1.08 | 12.18 | 1.58 |
| Cyclotetrasiloxane, octamethyl- | 2,2,4,4,6,6,8,8-octamethyl-1,3,5,7,2,4,6,8-tetraoxatetrasilocane | C[Si]1(O[Si](O[Si](O[Si](O1)(C)C)(C)C)(C)C)C | 11169 | 296.61  [C_8_H_24_O_4_Si_4_](https://pubchem.ncbi.nlm.nih.gov/#query=C8H24O4Si4) | 1.5 | 13.448 | 3.01 |
| Phosphonoacetic Acid, 3TMS derivative | trimethylsilyl 2-bis(trimethylsilyloxy)phosphorylacetate | C[Si](C)(C)OC(=O)CP(=O)(O[Si](C)(C)C)O[Si](C)(C)C | 631032 | 356.57   \| [C_11_H_29_O_5_PSi_3_](https://pubchem.ncbi.nlm.nih.gov/#query=C11H29O5PSi3) \| \| --- \| | 0.75 | 13.703 | 1.5 |
| Phenol, 2-chloro-5-methyl- | 2-chloro-5-methylphenol | CC1=CC(=C(C=C1)Cl)O | 12008 | 142.58  [C_7_H_7_ClO](https://pubchem.ncbi.nlm.nih.gov/#query=C7H7ClO) | 0.65 | 14.709 | 1.09 |
| Cyclohexasiloxane, dodecamethyl- | 2,2,4,4,6,6,8,8,10,10,12,12-dodecamethyl-1,3,5,7,9,11-hexaoxa-2,4,6,8,10,12-hexasilacyclododecane | C[Si]1(O[Si](O[Si](O[Si](O[Si](O[Si](O1)(C)C)(C)C)(C)C)(C)C)(C)C)C | 10911 | 444.92 [C_12_H_36_O_6_Si_6_](https://pubchem.ncbi.nlm.nih.gov/#query=C12H36O6Si6) | 0.76 | 14.839 | 1.18 |
| Cyclopentasiloxane, decamethyl- | 2,2,4,4,6,6,8,8,10,10-decamethyl-1,3,5,7,9,2,4,6,8,10-pentaoxapentasilecane | C[Si]1(O[Si](O[Si](O[Si](O[Si](O1)(C)C)(C)C)(C)C)(C)C)C | 10913 | 370.77 [C_10_H_30_O_5_Si_5_](https://pubchem.ncbi.nlm.nih.gov/#query=C10H30O5Si5) | 0.76 | 15.67 | 1.45 |
| 3,3,5-Triethoxy-1,1,1,7,7,7-hexamethyl-5-(trimethylsilyloxy)tetrasiloxane | [diethoxy(trimethylsilyloxy)silyl] ethyl bis(trimethylsilyl) silicate | CCO[Si](OCC)(O[Si](C)(C)C)O[Si](OCC)(O[Si](C)(C)C)O[Si](C)(C)C | 553849 | 474.91  [C_15_H_42_O_7_Si_5_](https://pubchem.ncbi.nlm.nih.gov/#query=C15H42O7Si5) | 0.49 | 15.935 | 0.87 |
| Phenol, 2,6-bis(1,1-dimethylethyl)- | 2,6-di*tert*-butylphenol | CC(C)(C)C1=C(C(=CC=C1)C(C)(C)C)O | 31405 | 206.32  [C_14_H_22_O](https://pubchem.ncbi.nlm.nih.gov/#query=C14H22O) | 0.56 | 16.902 | 0.94 |
| Cycloheptasiloxane, tetradecamethyl- | 2,2,4,4,6,6,8,8,10,10,12,12,14,14-tetradecamethyl-1,3,5,7,9,11,13-heptaoxa-2,4,6,8,10,12,14-heptasilacyclotetradecane | C[Si]1(O[Si](O[Si](O[Si](O[Si](O[Si](O[Si](O1)(C)C)(C)C)(C)C)(C)C)(C)C)(C)C)C | 7874 | 519.07  [C_14_H_42_O_7_Si_7_](https://pubchem.ncbi.nlm.nih.gov/#query=C14H42O7Si7) | 0.79 | 17.199 | 1.34 |
| 2,5-Cyclohexadiene-1,4-dione, 2,6-bis(1,1-dimethylethyl)- | 2,6-di*tert*-butylcyclohexa-2,5-diene-1,4-dione | CC(C)(C)C1=CC(=O)C=C(C1=O)C(C)(C)C | 12867 | 220.31  [C_14_H_20_O_2_](https://pubchem.ncbi.nlm.nih.gov/#query=C14H20O2) | 0.63 | 17.291 | 1.18 |
| 1-Dodecanol | dodecan-1-ol | CCCCCCCCCCCCO | 8193 | 186.33  [C_12_H_26_O](https://pubchem.ncbi.nlm.nih.gov/#query=C12H26O) or CH_3_(CH_2_)_10_CH_2_OH | 0.78 | 17.358 | 1.27 |
| Cyclohexasiloxane, dodecamethyl- | 2,2,4,4,6,6,8,8,10,10,12,12-dodecamethyl-1,3,5,7,9,11-hexaoxa-2,4,6,8,10,12-hexasilacyclododecane | C[Si]1(O[Si](O[Si](O[Si](O[Si](O[Si](O1)(C)C)(C)C)(C)C)(C)C)(C)C)C | 10911 | 444.92  [C_12_H_36_O_6_Si_6_](https://pubchem.ncbi.nlm.nih.gov/#query=C12H36O6Si6) | 1.04 | 17.9 | 1.41 |
| o-Hydroxybiphenyl | 2-phenylphenol | C1=CC=C(C=C1)C2=CC=CC=C2O | 7017 | 170.21  [C_12_H_10_O](https://pubchem.ncbi.nlm.nih.gov/#query=C12H10O) or C_6_H_5_C_6_H_4_OH | 1.1 | 18.065 | 1.02 |
| 2,2,4-Trimethyl-1,3-pentanediol diisobutyrate | [2,2,4-trimethyl-3-(2-methylpropanoyloxy)pentyl] 2-methylpropanoate | CC(C)C(C(C)(C)COC(=O)C(C)C)OC(=O)C(C)C | 23284 | 286.41   \| [C_16_H_30_O_4_](https://pubchem.ncbi.nlm.nih.gov/#query=C16H30O4) \| \| --- \| | 0.55 | 18.987 | 0.85 |
| 2,6-Bis(1,1-dimethylethyl)-4-(1-oxopropyl)phenol | 1-(3,5-di*tert*-butyl-4-hydroxyphenyl)propan-1-one | CCC(=O)C1=CC(=C(C(=C1)C(C)(C)C)O)C(C)(C)C | 616172 | 262.4   \| [C_17_H_26_O_2_](https://pubchem.ncbi.nlm.nih.gov/#query=C17H26O2) \| \| --- \| | 4.79 | 19.528 | 5.34 |
| Decanoic acid, decyl ester | decyl decanoate | CCCCCCCCCCOC(=O)CCCCCCCCC | 74247 | 312.5  [C_20_H_40_O_2_](https://pubchem.ncbi.nlm.nih.gov/#query=C20H40O2) | 0.4 | 19.806 | 0.59 |
| Cycloheptasiloxane, tetradecamethyl- | 2,2,4,4,6,6,8,8,10,10,12,12,14,14-tetradecamethyl-1,3,5,7,9,11,13-heptaoxa-2,4,6,8,10,12,14-heptasilacyclotetradecane | C[Si]1(O[Si](O[Si](O[Si](O[Si](O[Si](O[Si](O1)(C)C)(C)C)(C)C)(C)C)(C)C)(C)C)C | \| 7874 \| \| --- \| | 519.07  [C_14_H_42_O_7_Si_7_](https://pubchem.ncbi.nlm.nih.gov/#query=C14H42O7Si7) | 0.77 | 20.306 | 1.04 |
| 1,4-Benzenediol, 2,5-bis(1,1-dimethylethyl)- | 2,5-di*tert*-butylbenzene-1,4-diol | CC(C)(C)C1=CC(=C(C=C1O)C(C)(C)C)O | \| 2374 \| \| --- \| | 222.32   \| C_14_H_22_O_2_ \| \| --- \| | 1.6 | 20.396 | 1.34 |
| 5-Isopropyl-2-methylphenethyl acetate | 2-(2-methyl-5-propan-2-ylphenyl)ethyl acetate | CC1=C(C=C(C=C1)C(C)C)CCOC(=O)C | 589435 | 220.31  [C_14_H_20_O_2_](https://pubchem.ncbi.nlm.nih.gov/#query=C14H20O2) | 0.56 | 20.648 | 0.69 |
| Cyclononasiloxane, octadecamethyl- | 2,2,4,4,6,6,8,8,10,10,12,12,14,14,16,16,18,18-octadecamethyl-1,3,5,7,9,11,13,15,17-nonaoxa-2,4,6,8,10,12,14,16,18-nonasilacyclooctadecane | C[Si]1(O[Si](O[Si](O[Si](O[Si](O[Si](O[Si](O[Si](O[Si](O1)(C)C)(C)C)(C)C)(C)C)(C)C)(C)C)(C)C)(C)C)C | 11172 | 667.4  [C_18_H_54_O_9_Si_9_](https://pubchem.ncbi.nlm.nih.gov/#query=C18H54O9Si9) | 0.83 | 21.973 | 1.3 |
| Morpholine, 4-octadecyl- | 4-octadecylmorpholine | CCCCCCCCCCCCCCCCCCN1CCOCC1 | 85475 | 339.6   \| [C_22_H_45_NO](https://pubchem.ncbi.nlm.nih.gov/#query=C22H45NO) \| \| --- \| | 0.46 | 24.014 | 0.59 |
| Phenol, 3,5-bis(1,1-dimethylethyl)- | 3,5-ditert-butylphenol | CC(C)(C)C1=CC(=CC(=C1)O)C(C)(C)C | [70825](https://pubchem.ncbi.nlm.nih.gov/compound/70825) | \| 206.32 [[C_14_H_22_O](https://pubchem.ncbi.nlm.nih.gov/#query=C14H22O)](https://pubchem.ncbi.nlm.nih.gov/#query=C17H26O2) \| \| --- \| | 2.98 | 17.824 | 6.24 |
| Dimethyl palmitamine | *N*,*N*-dimethylhexadecan-1-amine | CCCCCCCCCCCCCCCCN(C)C | 16221 | 269.5  [C_18_H_39_N](https://pubchem.ncbi.nlm.nih.gov/#query=C18H39N) | 0.6 | 24.353 | 0.67 |
| 1H-Inden-5-ol, 2,3-dihydro-1,1,3,3-tetramethyl-4,6-bis(1-methylethyl)- | 1,1,3,3-tetramethyl-4,6-di(propan-2-yl)-2*H*-inden-5-ol | CC(C)C1=CC2=C(C(=C1O)C(C)C)C(CC2(C)C)(C)C | \| 621162 \| \| --- \| | 274.4   \| [C_19_H_30_O](https://pubchem.ncbi.nlm.nih.gov/#query=C19H30O) \| \| --- \| | 1.16 | 24.459 | 1.11 |
| Cyclodecasiloxane, eicosamethyl- | 2,2,4,4,6,6,8,8,10,10,12,12,14,14,16,16,18,18,20,20-icosamethyl-1,3,5,7,9,11,13,15,17,19-decaoxa-2,4,6,8,10,12,14,16,18,20-decasilacycloicosane | C[Si]1(O[Si](O[Si](O[Si](O[Si](O[Si](O[Si](O[Si](O[Si](O[Si](O1)(C)C)(C)C)(C)C)(C)C)(C)C)(C)C)(C)C)(C)C)(C)C)C | 519601 | 741.5 [C_20_H_60_O_10_Si_10_](https://pubchem.ncbi.nlm.nih.gov/#query=C20H60O10Si10) | 2.6 | 24.55 | 1.11 |
| Hexadecanoic acid, methyl ester | methyl hexadecanoate | CCCCCCCCCCCCCCCC(=O)OC | \| 8181 \| \| --- \| | 270.5  [C_17_H_34_O_2_](https://pubchem.ncbi.nlm.nih.gov/#query=C17H34O2) | 1.2 | 24.683 | 1.45 |
| n-Hexadecanoic acid | hexadecanoic acid | CCCCCCCCCCCCCCCC(=O)O | \| 985 \| \| --- \| | 256.42  [C_16_H_32_O_2_](https://pubchem.ncbi.nlm.nih.gov/#query=C16H32O2) | 13.02 | 25.372 | 7 |
| Cyclononasiloxane, octadecamethyl- | 2,2,4,4,6,6,8,8,10,10,12,12,14,14,16,16,18,18-octadecamethyl-1,3,5,7,9,11,13,15,17-nonaoxa-2,4,6,8,10,12,14,16,18-nonasilacyclooctadecane | C[Si]1(O[Si](O[Si](O[Si](O[Si](O[Si](O[Si](O[Si](O[Si](O1)(C)C)(C)C)(C)C)(C)C)(C)C)(C)C)(C)C)(C)C)C | \| 11172 \| \| --- \| | 667.4  [C_18_H_54_O_9_Si_9_](https://pubchem.ncbi.nlm.nih.gov/#query=C18H54O9Si9) | 1.2 | 25.964 | 1.02 |
| Cyclooctasiloxane, hexadecamethyl- | 2,2,4,4,6,6,8,8,10,10,12,12,14,14,16,16-hexadecamethyl-1,3,5,7,9,11,13,15-octaoxa-2,4,6,8,10,12,14,16-octasilacyclohexadecane | C[Si]1(O[Si](O[Si](O[Si](O[Si](O[Si](O[Si](O[Si](O1)(C)C)(C)C)(C)C)(C)C)(C)C)(C)C)(C)C)C | \| 11170 \| \| --- \| | \| 593.2 \| \| --- \|   [C_16_H_48_O_8_Si_8_](https://pubchem.ncbi.nlm.nih.gov/#query=C16H48O8Si8) | 3.77 | 27.521 | 3.52 |
| Cyclononasiloxane, octadecamethyl- | 2,2,4,4,6,6,8,8,10,10,12,12,14,14,16,16,18,18-octadecamethyl-1,3,5,7,9,11,13,15,17-nonaoxa-2,4,6,8,10,12,14,16,18-nonasilacyclooctadecane | C[Si]1(O[Si](O[Si](O[Si](O[Si](O[Si](O[Si](O[Si](O[Si](O1)(C)C)(C)C)(C)C)(C)C)(C)C)(C)C)(C)C)(C)C)C | 11172 | 667.4  [C_18_H_54_O_9_Si_9_](https://pubchem.ncbi.nlm.nih.gov/#query=C18H54O9Si9) | 3.08 | 30.377 | 3.53 |
| Heptasiloxane, hexadecamethyl- | bis[[[dimethyl(trimethylsilyloxy)silyl]oxy-dimethylsilyl]oxy]-dimethylsilane | C[Si](C)(C)O[Si](C)(C)O[Si](C)(C)O[Si](C)(C)O[Si](C)(C)O[Si](C)(C)O[Si](C)(C)C | 10912 | 533.1   \| [C_16_H_48_O_6_Si_7_](https://pubchem.ncbi.nlm.nih.gov/#query=C16H48O6Si7) \| \| --- \| | 0.85 | 30.53 | 0.94 |
| Cyclononasiloxane, octadecamethyl- | 2,2,4,4,6,6,8,8,10,10,12,12,14,14,16,16,18,18-octadecamethyl-1,3,5,7,9,11,13,15,17-nonaoxa-2,4,6,8,10,12,14,16,18-nonasilacyclooctadecane | C[Si]1(O[Si](O[Si](O[Si](O[Si](O[Si](O[Si](O[Si](O[Si](O1)(C)C)(C)C)(C)C)(C)C)(C)C)(C)C)(C)C)(C)C)C | 11172 | 667.4  [C_18_H_54_O_9_Si_9_](https://pubchem.ncbi.nlm.nih.gov/#query=C18H54O9Si9) | 3.2 | 33.17 | 3.34 |
| Heptasiloxane, hexadecamethyl- | bis[[[dimethyl(trimethylsilyloxy)silyl]oxy-dimethylsilyl]oxy]-dimethylsilane | C[Si](C)(C)O[Si](C)(C)O[Si](C)(C)O[Si](C)(C)O[Si](C)(C)O[Si](C)(C)O[Si](C)(C)C | \| 10912 \| \| --- \| | 533.1  [C_16_H_48_O_6_Si_7_](https://pubchem.ncbi.nlm.nih.gov/#query=C16H48O6Si7) | 0.7 | 33.523 | 0.78 |
| Cyclononasiloxane, octadecamethyl- | 2,2,4,4,6,6,8,8,10,10,12,12,14,14,16,16,18,18-octadecamethyl-1,3,5,7,9,11,13,15,17-nonaoxa-2,4,6,8,10,12,14,16,18-nonasilacyclooctadecane | C[Si]1(O[Si](O[Si](O[Si](O[Si](O[Si](O[Si](O[Si](O[Si](O1)(C)C)(C)C)(C)C)(C)C)(C)C)(C)C)(C)C)(C)C)C | \| 11172 \| \| --- \| | 667.4  [C_18_H_54_O_9_Si_9_](https://pubchem.ncbi.nlm.nih.gov/#query=C18H54O9Si9) | 3.48 | 36.564 | 2.82 |


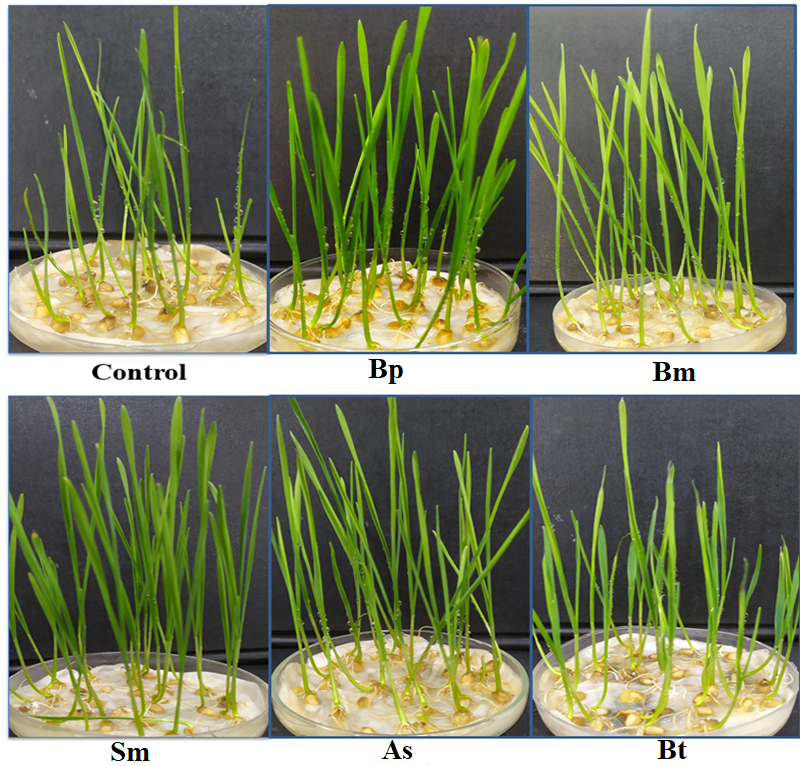


**Figure S1:** Petridis Growth of *Triticum aestivum* seeds treated with bacterial species in petri-dishes. Data were recorded after 7 days of inoculation. Here, Bp, Bm, Sm, As, and Bt indicates *Bacillus pseudomycoides, Bacillus massilioanrexius*, *Serratia marcescens*, *Acinetobacter* sp.*,* and *Bacillus thuringiensis* respectively.


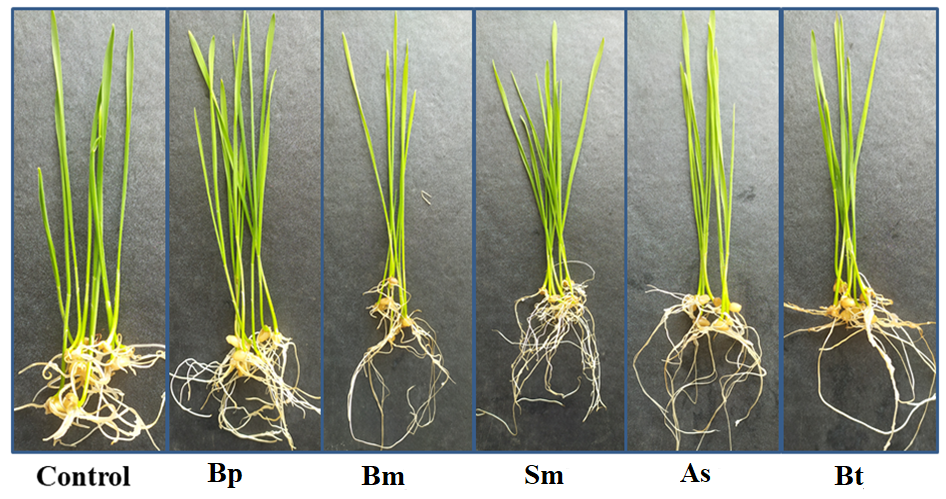


**Figure S2:** Root and shoot growth of *Triticum aestivum* treated with bacterial species. Here, Bp, Bm, Sm, As, and Bt indicate *Bacillus pseudomycoides, Bacillus massilioanrexius*, *Serratia marcescens*, *Acinetobacter* sp.*,* and *Bacillus thuringiensis* respectively. Data were recorded after 15 days of germination.

**
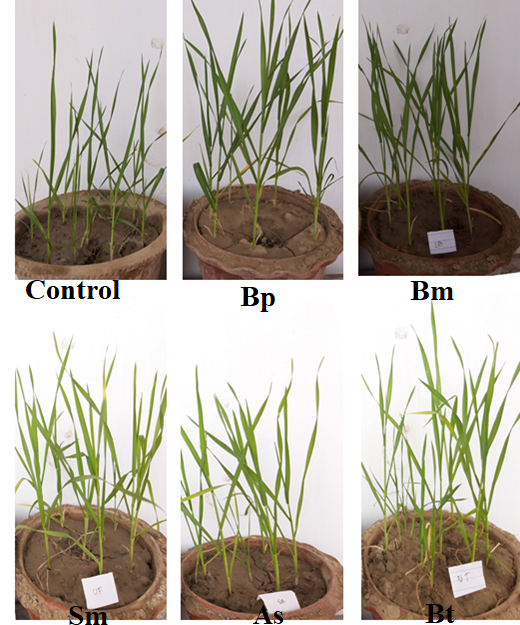
**

**Figure S3:** Morphological growth of *Triticum aestivum* seeds in pot conditions treated with bacterial species. Here, Bp, Bm, Sm, As, and Bt indicates *Bacillus pseudomycoides, Bacillus massilioanrexius*, *Serratia marcescens*, *Acinetobacter* sp.*,* and *Bacillus thuringiensis* respectively. Data were recorded after 15 days of germination.


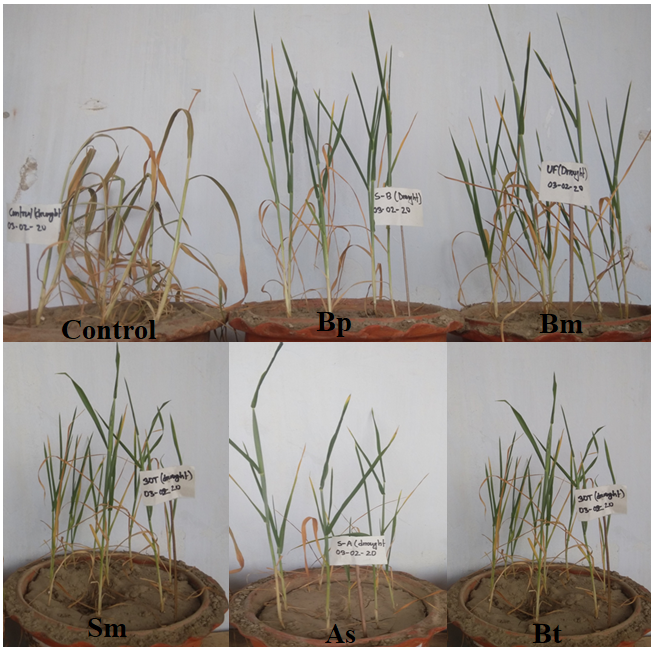


**Figure S4:** Morphological drought stress tolerance activity of *Triticum aestivum* seeds treated with bacterial species. Here, Bp, Bm, Sm, As, and Bt indicates *Bacillus pseudomycoides, Bacillus massilioanrexius*, *Serratia marcescens*, *Acinetobacter* sp.*,* and *Bacillus thuringiensis,* respectively. Data were recorded after 15 days of drought stress induction.


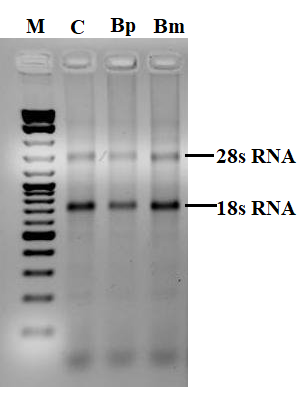


**Figure S5:** Agarose gel (1%) electrophoresis of extracted RNA after treated with DNase. Here, M indicates Marker; C indicates wheat sample without treatment; Bp and Bm indicate the wheat sample treated with *Bacillus pseudomycoides and Bacillus massilioanrexius*, respectively.
